# Sponge-Based Flow Control in Laminate Capillary-Driven Electrochemical Microfluidic Devices for Viscous Sample Analysis

**DOI:** 10.1021/acs.analchem.5c07262

**Published:** 2026-03-30

**Authors:** Diele. A. G. Araújo, Thaisa A. Baldo, Thiago R. L. C. Paixão, Charles S. Henry

**Affiliations:** a Institute of Chemistry, Department of Fundamental Chemistry, 28133 University of São Paulo, São Paulo, SP 05508-000, Brazil; b Department of Chemistry, 3447 Colorado State University, Fort Collins, Colorado 80523, United States; c School of Biomedical and Chemical Engineering, 3447 Colorado State University, Fort Collins, Colorado 80523, United States

## Abstract

Microfluidic systems are an attractive strategy for developing environmentally safer analytical methods and collecting real-time information to perform ″in loco″ analyses. Laminate capillary-driven microfluidic devices are a promising approach that can achieve fast results using low-cost devices. Despite significant advancements in capillary-driven microfluidic devices, analyzing viscous samples, particularly biological fluids, remains a challenge because the flow is highly dependent on the viscosity of the solution. Additionally, there has been a limited ability to control the flow rate. Herein, we proposed a laminate capillary-driven microfluidic device to overcome the flow issues where commercial sponges are used as passive pumps. A capillary-driven microfluidic device was coupled to electrochemical detection to provide quantitative results. After optimization, sponges provide sufficient control flow independent of the material type used to make the device. Additionally, the proposed microfluidic design enables analysis using higher viscosity solutions without compromising the electrochemical signal. The device was used to quantify paracetamol in undiluted human saliva using a generator-collector mode, with a recovery rate of nearly 100%. An important finding in this work was the possibility of analyzing viscous saliva without requiring any dilution. Additionally, the new design, which utilizes the sponge as a passive pump, opens the possibility of performing reactions (sample preparation steps) within the microfluidic channel, as the flow can be controlled by simply replacing the sponge. These results pave the way for the development of capillary-driven microfluidic devices with electrochemical detection for saliva analysis outside laboratory settings.

## Graphical abstract

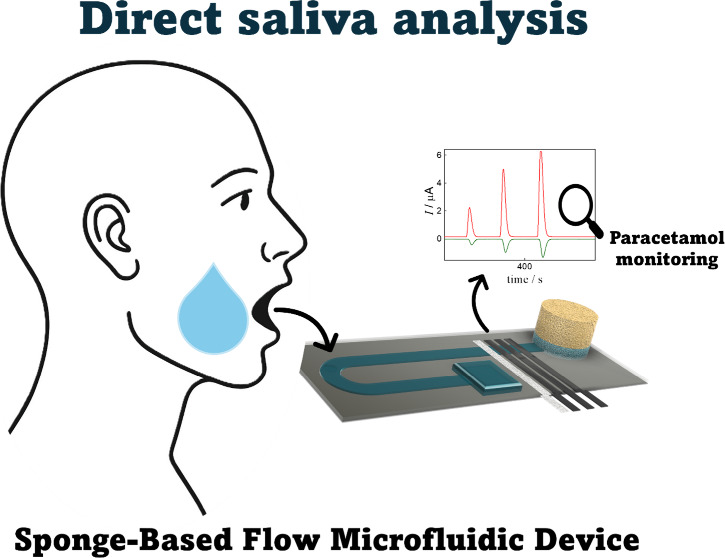

## Introduction

Microfluidic systems are a promising approach to developing integrated analysis platforms, such as lab-on-chips, that reproduce laboratory-scale processes in a miniaturized format.^1^ Silicon and glass were the first materials to be used in the manufacture of microfluidic devices.^2^ However, advancements in microfluidics have gradually replaced these materials with polymers such as polydimethylsiloxane (PDMS),^3^ paper,^4^ and plastic materials,^5^ which are less expensive, more accessible, and easier to machine, allowing for flow via capillary action. Capillary-driven devices enable flow manipulation without pumps, utilizing the surface tension of a fluid as it interacts with channel walls or fibers, as described in the case study.^6^ In this context, the development of microfluidic paper-based devices (μPADs), first reported by Whitesides’ Group, opened new opportunities in microfluidic research.^4^ μPAD’s ability to store reagents enables chemical and biochemical reactions within the device, drawing the attention of analytical researchers.

Fluid control in microfluidic devices is a critical factor for ensuring the reproducibility and reliability of experimental assays. The flow rate directly influences the physicochemical interactions and reaction kinetics occurring within the microchannels, as well as the accuracy of data acquisition. Therefore, establishing a system capable of regulating the flow is interesting for optimizing microfluidic performance and obtaining consistent experimental outcomes.^7,8^

More recently, lamination-based capillary-driven microfluidic devices have been proposed in the literature.^9–12^ In the lamination method, fluid flow is driven by capillary forces within the gap formed between two precut layers of paper or polymer films, aiming to overcome the limitations reached by capillary porous materials.^13^ The microchannel geometry is defined in each layer before alignment and bonding, allowing precise formation of the capillary-driven flow path.^14^ Different materials, such as polyester film, acrylic sheets, glass slides, paper, and transparency film, have been bonded using double-sided adhesive (DSA), ethylene-vinyl acetate, oxygen plasma, and/or toner.^15^ When compared with μPADs, the lamination-based capillary-driven devices present advantageous features, including fast and uniform flow, which minimizes the evaporation and allows reagents to be stored similarly to those in μPADs.^8,13^ Additionally, push/burst valve systems can be easily implemented on lamination-based capillary devices, allowing for the flow to be stopped and opened.^13^

Despite significant advancements in capillary-driven microfluidic devices, analyzing viscous samples, particularly biological fluids, is challenging since the viscosity of the solution strongly influences the flow. Various efforts have been made to address this issue, including modifications to the channel and inlet geometry,^13^ variations in size and height,^14^ the hydrophobicity of the materials used in the layers, and device designs,^13^ evaporation pump,^16^ push and burst valve pump,^13^ paper plug strategies.^17^ Moreover, different pumping materials, such as hydrogels and 2.5*D*/3D microfabricated pumps, have been investigated to enable the transition of microfluidic devices from laboratory prototypes to low-cost, user-friendly point-of-care systems.^18,19^ Nevertheless, the paper continues to be widely used as a passive pump and/or waste pad, which remains responsible for controlling the flow in these systems.^12,13,20,21^ Capillary action in paper decreases over time, reducing flow rate and control during analysis and requiring frequent device replacements, which can affect flow and reduce signal reproducibility. Therefore, exploring new substrate materials is necessary, particularly those capable of integrating the functions of both waste pads and passive pumps, to enhance flow control and broaden the applicability of capillary-driven microfluidic systems. Although extensive research has been conducted to improve fluid flow in microfluidic systems,^17,22^ its application to lamination-based capillary devices is still limited. Furthermore, to our knowledge, no studies have investigated the use of alternative materials as passive pumps to enhance flow control in lamination-based capillary devices.

In this context, sponges are an interesting material for passive pump applications due to their specific features, such as liquid wicking and the capacity to be reused after simple mechanical stress, in addition to being low-cost.^23^ Sponges can be produced from various materials, including natural options like sea sponges and synthetic ones such as polyurethane (PU), polyester, and cellulose. These materials enable a wide range of applications, including microfluidic solution sampling integrated with ion-selective electrodes,^23^ the removal of micropollutants,^24^ and a disposable pressure pump for microfluidic lab-on-a-chip systems.^25^ Although some applications of sponges have been reported in the literature, to the best of our knowledge, their use as a passive pump to control flow in microfluidic devices has not been previously described.

Herein, we proposed a lamination-based capillary-driven microfluidic device to overcome flow issues using commercial sponges as passive pumps, enabling the direct analysis of undiluted saliva. Saliva has attracted growing interest as a promising biological fluid for disease diagnostics due to the presence of a wide range of biomarkers.^26^ Many of these biomarkers are found at concentrations comparable to those in blood, making saliva a valuable alternative for clinical analysis. Additionally, saliva collection is a noninvasive, stress-free, simple, and rapid procedure to perform. It can also be performed without clinician training.^27^

In this study, we performed a systematic optimization of laminate-based devices using different materials to assemble them, and the flow was controlled by commercial sponges set at the outlet. Flow performance was evaluated in terms of velocity and electrochemical signal response. Analytical curves for nicotine, dopamine, and paracetamol were constructed in 0.5% sodium carboxymethyl cellulose (SCMC), simulating the viscosity of saliva. The versatility of the device was evaluated in electrochemical generator–collector (GC) mode, where two distinct potentials were applied to quantify paracetamol in undiluted saliva. A key innovation of this device is its ability to analyze viscous saliva samples directly without the need for dilution or protein precipitation. Furthermore, the use of a sponge as a passive pump introduces the potential to better control flow and perform in-channel reactions or sample preparation steps by simply exchanging the pump material.

## Experimental Section

### Chemicals and Materials

All solutions were prepared using deionized Milli-Q water (18.2 MΩ ·cm) from Millipore Sigma, USA. All chemicals (analytical grade) were used without additional purification. Aqueous solutions with 10 and 20 wt % glycerin were used to evaluate the flow. Sodium carboxymethylcellulose - SCMC in the range of 0.25, 0.50, 0.75, 1.00, 1.25, and 1.50% (w/v) was used to mimic human saliva viscosity.^28^ Blue food dye solution was used to measure the flow velocity. Potassium chloride and potassium ferrocyanide were used as the probe for electrochemical characterizations. Phosphate buffer saline (PBS) composed of 140 mmol L^–1^ sodium chloride and 2.7 mmol L^–1^ potassium chloride at pH 7.4 was prepared by dissolving a tablet in 500 mL of deionized water. The stock solutions of Nicotine - NIC, Dopamine – DOP, and acetaminophen or paracetamol – PAR were prepared in PBS. All chemicals were purchased from Sigma-Aldrich (St. Louis, Missouri), except the PBS, which was purchased from Research Products International, USA.

### Laminate capillary-driven microfluidic electrochemical devices

Capillary-driven microfluidic devices were fabricated using laminating double-sided adhesive - DSA 467MP and 468MP, with 75 and 100 μm thickness, respectively, both acquired from 3M, and two types of transparency films: 9984, and PET films 9962, both with 99 μm thickness, obtained from 3 M (MN, USA). The devices were designed with two inlets coupled to the straight channel (3 mm-width and 70 mm-length): one inlet worked as an electrolyte (carrier fluid) reservoir (200 or 500 μL), while the second inlet was used as a sample injection (2 mm diameter), and one outlet (25 mm diameter) to set the passive pump. The use of a larger volume created an additional pumping force due to hydrostatic and gravity forces. The curve design was fabricated using three layers, with the straight channel for the curve channel being the only modification, as shown in Figure S1. Two sponges with different materials were studied as waste pad/passive pumps: fiber/resin and cellulose, chosen based on their commercial availability. By using commercially available parts, we can reduce costs, making it easier to develop low-cost microfluidic devices with higher reproducibility. For this, these sponges were cut in a circular format with a 25 mm diameter, 5 mm fiber/resin, and 15 mm height for cellulose. A three-electrode and/or generation-collection system was screen-printed on transparency film (bottom layer) using carbon ink (Cl-2057, Engineered Materials Solutions, Inc.) and a mesh screen, and dried at 60 °C for 30 min. For analytical applications (nicotine, dopamine, and paracetamol), a thin layer was applied to the reference electrode using Ag|AgCl ink (Sigma-Aldrich) and cured at 60 °C (∼10 min). Figure 1 shows the schematic representation of the capillary microfluidic device coupled to the electrochemical detection system in the evaluated designs.

**Figure 1:**
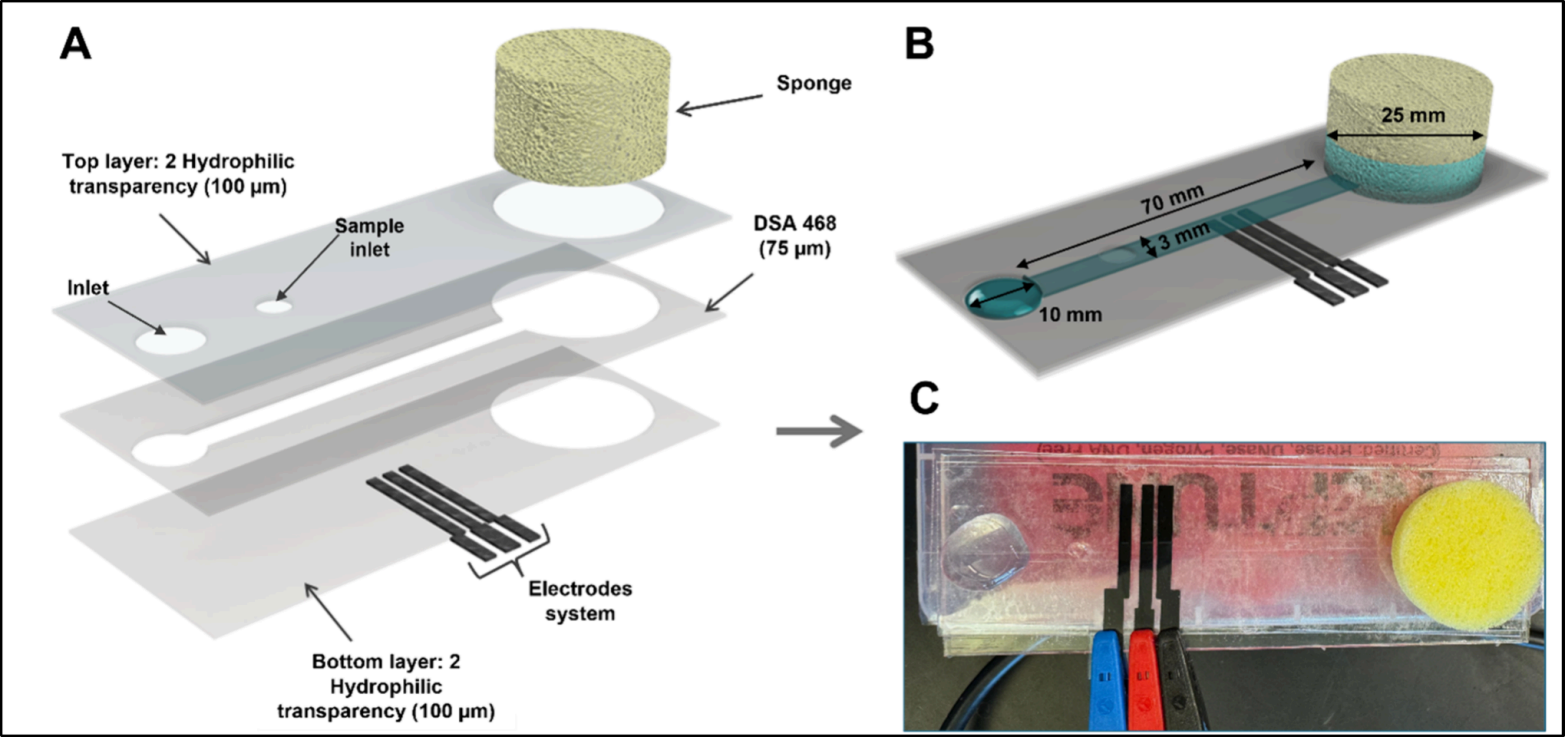
(A, B) Schematic representation of the proposed sponge microfluidic device. (C) Real image of the proposed device.

All measurements were performed at approximately 25 °C and 30% relative humidity. The colorimetric assays using a dye solution were recorded using a portable camera (iPhone 15 Pro, Apple) under the lab lighting environment. The electrochemical experiments were recorded using a portable bipotentiostat (EmStat4s-PalmSens) controlled by PsTrace5.9.

### Analytical Performance and Human Saliva Analysis

For all measurements (colorimetric and electrochemical), 200 μL of the carrier solution were pipetted into the inlet reservoir before analysis. 4 μL of the dye/analyte/sample solutions were injected through a sample hole on the top layer, 20 mm from the inlet. The flow velocity, depending on the sponge used, was achieved by injecting the dye solution (4 μL) and was measured along a 40 mm section of the channel. The details of the measurements are shown in the Supplementary Video 1. The analytical signal was used; the current intensity was obtained from the peak height measured from the peak baseline to the peak apex. At the same time, the peak area was calculated from the integration of the peak. The saliva was collected from nonidentified individuals using the passive drool collection method, approximately 2 mL, under Institutional Review Board approval. The individuals had been fasting for at least 3 h before the collection. The quantification of paracetamol in saliva was performed using a curved-channel (see Figure S1) design to demonstrate the versatility of the device, which could be configured in different ways. Also, a generator-collector (GC) electrochemical system was implemented to minimize interference effects.

## Results And Discussion

### Sponge Pumping

To evaluate the use of a sponge as a passive pump, the laminate capillary microfluidic device was assembled according to the Experimental section, using different sponge materials as passive pumps. Figure 2A shows the representation and a real image of the devices studied. Figure 2B, as shown in Supplementary Video 1, indicates that flow velocity depends on the sponge material used as the waste pad, suggesting that the sponge can also function as a passive pump. A faster flow was observed for the cellulose substrate than for the fiber/resin, at (4.61 ± 0.38) and (1.28 ± 0.17) mm s^–1^, respectively. These values can be attributed to the pumping action established in each sponge used, with cellulose exhibiting greater wettability than fiber. As expected, the flow velocity achieved for all sponges is faster than reported for a microfluidic device fabricated using a paper substrate (Table S1).^15,29,30^ The maximum number of injections achievable using a single sponge was evaluated. Successive injections of 4 μL of dye solution were performed, and the flow velocity was measured. As shown in Figure S2, the velocity gradually decreased with successive injections, with reductions of 24% and 31% after 40 injections for the cellulose and fiber sponges, respectively. These results indicate progressive sponge saturation. Despite this decrease, the electrochemical signal was not compromised, as shown in the results presented in this work. However, for the proposed application, reproducibility is more important than maximum sponge capacity, since the main goal is to develop a single-use device for viscous solutions, as will be demonstrated in the generator-collector mode for paracetamol sensing in human saliva section.

**Figure 2:**
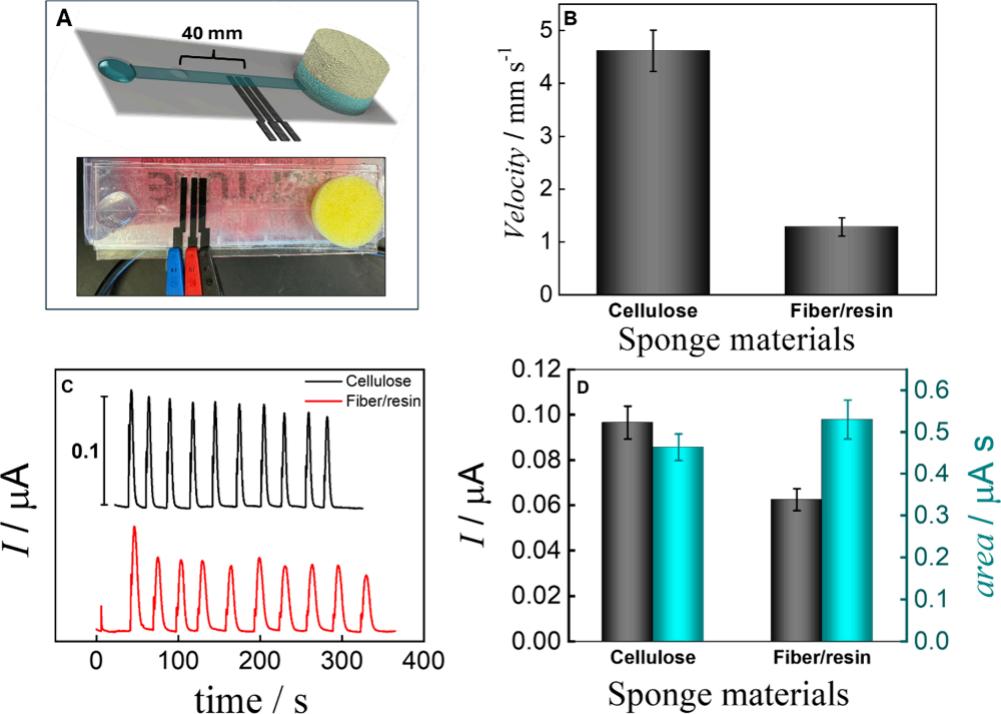
(A) Schematic representation and image of the device. (B) Velocity obtained using the straight channel coupled with different sponge materials as passive pumps. (C) Transient current signals for injections of 4 μL 5 mmol L^–1^ [Fe­(CN)_6_]^3–/4–^ in 0.5 mol L^–1^ KCl using different passive pump sponges: cellulose (black line); fiber/resin (red line). (D) I_p_ and area as a function of the passive pump. Carrier solution: 0.5 mol L^–1^ KCl. E_det_ = +0.35 V vs carbon pseudo-RE. Bar errors show the average and standard deviations of 10 injections.

Regarding the lamination-based devices, there are a few studies that provide information about the flow features that limit comparison. Thus, these findings provide important insights into characterizing the flow in lamination-based microfluidic devices, suggesting that flow velocity can be effectively controlled to the desired flow rate by simply selecting the passive pump material at the channel outlet, as applicable. This approach eliminates the need for external mechanical pumps commonly used in conventional microfluidic systems and addresses the flow limitations of the paper used as the waste pad.

The influence of the sponge on the electrochemical signal was investigated by setting up a three-electrode system on the bottom layer of the microfluidic device, as previously introduced by the Henry group,^12^ as shown in Figure 2A. In this case, the inlet was filled with 0.5 mol L^–1^ KCl, and 5.0 mmol L^–1^ potassium ferricyanide (III) was used as the electrochemical probe. The results presented in Figure 2C,D indicate that the signal was affected by the type of sponge studied. For cellulose sponges, we observed higher and narrower signals, confirming a faster flow rate with a peak time close to (13.5 ± 1.3) seconds.

The sponge controls flow by serving as a high-capacity capillary pump at the outlet. Once the straight laminate channel is filled, the pressure drop and the flow rate are determined mostly by the liquid uptake into the sponge rather than by the channel geometry or surface chemistry of the laminates.^31^ Capillary forces in the interconnected pores of the sponge generate a sustained suction that draws fluid through the channel; hydrostatic pressure from the inlet reservoir and gravity contribute.^32^ The structural parameters of the sponge modulate capillary pumping by increasing porosity, thereby increasing the available capillary surface area and total liquid uptake capacity. Optimal moderate pore sizes balance high capillary pressure from small pores with low hydraulic resistance from larger pores, since pores that are too small increase viscous drag and reduce flow rate, while pores that are too large generate insufficient capillary pressure. Higher wettability, lower contact angle θ, strengthens liquid–wall interactions and increases both initial imbibition and steady-state flow. This explains why the more hydrophilic cellulose sponge produced significantly faster, better-resolved transient signals than the fiber/resin sponge and why flow velocity remained almost constant across different channel materials but changed systematically with sponge material and solution viscosity.^31,32^

Although fiber sponges provide broader peaks than cellulose, the flow of the fiber sponges can also be considered fast, with a peak time of around (20.9 ± 2.2) seconds. As expected, the peak area increased when the flow rate decreased. However, this does not present a limitation since the reproducible transient signals demonstrate that a continuous flow was maintained throughout the experiments for all sponge types. This suggests that the waste pad/passive pump provides stable flow conditions by capillary action, making it suitable for electrochemical analyses. Since flow is primarily a parameter that affects the electrochemical signal, this suggests that the pump is a promising alternative for this purpose. The cellulose sponge was selected for the following experiments due to its faster flow.

### Lamination-Based Electrochemical Microfluidic Device Optimizations

Previous reports have shown that both the materials used and the design of the system affect the performance of lamination-based microfluidic devices.^10,13^ Thus, we optimized the system in terms of electrode positions, the distance between injection and detection, transparency, adhesive type, the number of layers, and inlet volume based on flow characteristics measured through electrochemical experiments and dye solution experiments. + 0.35 V (vs carbon) was chosen as the potential detection based on the cyclic voltammogram recorded in static mode, as shown in Figure S3. The working electrodes and reference electrodes were maintained at the same distance to prevent alterations in resistance.

#### Electrode Positions on the Microfluidic Device

To evaluate the electrode positions, we adjusted the position of the reference electrode during this optimization. As expected, the position of the reference electrode influences the electrochemical signal. When the pseudoreference electrode is placed at the beginning (configuration 1) or in the middle (configuration 2) of the electrode system on a microfluidic device (Figure S4), similar current intensities are observed. Although configuration 3, where the pseudoreference electrode is placed as the last electrode in the system, produces a higher current signal, this configuration achieves poor reproducibility, with an RSD of 20%, compared to 8% for configurations 1 and 2, calculated from the 10^th^ injection across three different devices. Therefore, configuration 3 is not suitable for assembling the microfluidic device. This behavior aligns with the most common microfluidic electrochemical systems, in which the reference electrode is set first to prevent interference from reaction products and intermediates that could alter the reference potential and impact accuracy.^7,29,30,33^ Based on these results, configuration 1 was used for further experiments.

#### Analytical Path

Next, the distance between the injection and the working electrode (analytical path) was evaluated. Interestingly, more intense amperometric peaks were observed as the length of the analytical path increased up to 30 mm, then decreased slightly, as shown in Figure S5. These results suggest that the stabilized flow from the sponge pump minimizes the sample dispersion over longer paths, unlike the conventional paper-based microfluidics devices.^30,34^

#### Channel Height

Another parameter evaluated was the channel height, which influences the flow in capillary microfluidic devices.^13,15,35^ To assess the impact of channel height, two DSA types were tested: DSA 468 and DSA 467, with heights of 75 and 50 μm, respectively. As shown in Figure 3A, there is no significant influence on the peak profile when the different channel heights were evaluated. Additionally, the results indicate that sponge pumping provides effective flow control, as flow velocity remained unaffected by channel height (Figure 3A). The 50 μm channel provided a slight increase in the electrochemical signal of approximately 14%, as shown in Figure S6. However, due to the challenges associated with assembling the device using DSA467 (which is thinner and more fragile), the 75 μm channel (DSA 468) was selected for its reproducibility and ease of handling during the assembly process.

**Figure 3:**
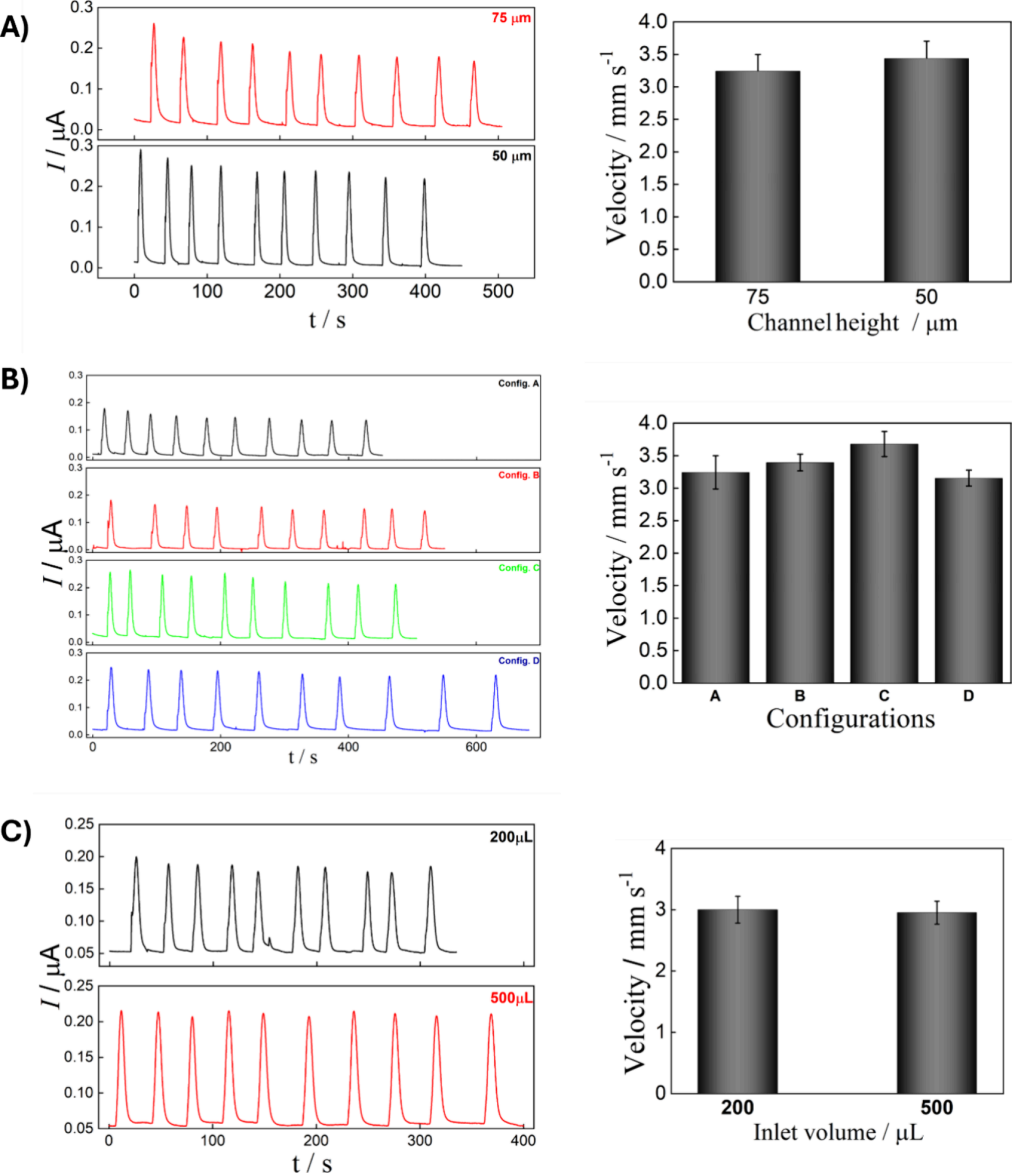
Influence of the channel height, transparency type, and inlet volume on the electrochemical response and flow velocity. (A) Transient current signals and respective flow velocity of devices assembled with channel heights of 75 and 50 μm. (B) Transient current signals and respective flow velocity of the different configuration arrays evaluated. (C) Transient current signals and respective flow velocity of the inlet volume. Amperometric measurements were conducted with injections of 5 μL 5 mmol L^–1^ [Fe­(CN)_6_]^3–/4–^ in 0.5 mol L^–1^ KCl. The flow velocity was calculated based on the time it took for a dye solution to travel through the straight channel. Carrier solution: 0.5 mol L^–1^ KCl, E_det_ = +0.35 V vs carbon pseudo-RE, passive pump: cellulose sponge, distance Inj-WE: 30 mm. Error bars show the average and standard deviations of 10 injections.

#### Types of Transparency Films

In the next step, we studied two types of transparency films: 9984 and 9962 PET films, named Transparency 1 and Transparency 2, respectively. Transparency 1 exhibits two distinct surfaces: one more hydrophobic, with a contact angle of 74.6 ± 0.5°, and one more hydrophilic, with a contact angle of 14.5 ± 2.7°, as demonstrated by the contact angle measurements presented in Figure S7. In contrast, both sides of Transparency 2 are hydrophilic, exhibiting contact angles of 28.1 ± 1.7° and 25.2 ± 0.4°, respectively (Figure S7). To evaluate the influence of each type of transparency, we established four combinations, as outlined in Table 1.

**Table 1:** Arrays Evaluated to Make the Microfluidic Devices Using Two Different Transparencies

	transparency		
configuration	bottom	top	RSD^a^ (%)
A	1 (hydrophobic)	1 (hydrophilic)	13
B	2 (hydrophilic)	1 (hydrophilic)	10
C	1 (hydrophobic)	2 (hydrophilic)	7
D	2 (hydrophilic)	2 (hydrophilic)	5

a Relative standard deviation obtained for the 10th injection in three different assembled devices.

As shown in Figure 3B, the peak profile remained consistent regardless of the type of transparency used during device assembly. There was no significant difference in flow dynamics, as the fluid velocity was not notably affected (see Figure 3B). These findings confirm that the flow is entirely driven by the sponge once the channel is filled, eliminating any influence from the transparency type, even when the hydrophobic side is used to fabricate the device. Jahanshahi-Anbuhi et al.^36^ also found that the transparency type (i.e., different contact angles) does not influence the flow depending on the liquid. A slight increase in signal intensity was observed for configurations C and D, 12% and 17%, respectively, when compared to configuration A (Figure S8). Therefore, configuration D was chosen for further testing, as it facilitates easier sensor printing and resulted in a lower RSD across three different assembled sensors (Table 1), indicating that, despite being manually assembled, the fabrication process is reproducible.

#### Inlet volume

Sequentially, two inlet volumes, 200 and 500 μL, were evaluated, both with a height of 175 μm, formed by one 75 μm DSA layer and one 100 μm transparency film. For the device with a 500 μL inlet, five layers were required to compensate for leakage between layers caused by hydrostatic pressure. One of these layers worked as an inlet connection and flow-control channel to the main channel (Figure S9). Although the electrochemical signal increased by 14% with the increase in inlet volume (Figure S10), this behavior is typically observed in microfluidic devices. However, this result should be attributed to the printed sensor rather than the flow characteristics since the velocity remained constant for both evaluated volumes (Figure 3C), averaging approximately 3.0 mm s^–1^, regardless of the materials or inlet volumes tested. The total flow time required to re-establish the baseline after each injection was around 16 s, significantly shorter than that reported for other laminate-capillary microfluidic devices using paper waste pads for pumping.^30,37^ Additionally, the proposed microfluidic device demonstrated a stable electrochemical response, as evidenced by unchanged peak profiles throughout the analysis. This consistency indicates that the system operated without flow interruptions or air entrapment issues.

Thus, after all optimizations, the selected optimal conditions were: first reference position, transparency type 2, an injection-working electrode distance of 30 mm, a channel height of 75 μm, and an inlet volume of 200 μL. Table S2 summarizes the evaluated and selected optimization conditions.

### Influence of the Solution Viscosity on the Passive Pump

The solution viscosity has an impact on the flow velocity in a microfluidic device. Under the optimized conditions, to evaluate the effect of solution viscosity, we used a straight microfluidic channel with a height of 75 μm and a length of 70 mm, connected to a 200 μL inlet reservoir. A 6 μL volume of the [Fe­(CN)_6_]^3–^/^4–^ or dye solution, prepared in the same medium as the carrier solution, was injected through the sample inlet. The viscosities of the solutions were adjusted by directly adding Glycerin. Table S3 shows the viscosity values for each solution studied.^13,38^ As previously reported, flow velocity decreases with increasing viscosity.^13^ This behavior can be attributed to increased fluid resistance resulting from higher internal friction. Also, more viscous solutions tend to exhibit lower surface tension, which further contributes to the reduction in flow velocity.^13^

In our study, the flow velocity remained nearly unchanged for pure water and the KCl, even when the concentration increased from 0.5 molL^–1^ to 1.0 molL^–1^, since and the peak times were the same, around 16 s. Also, this statement is supported by the consistent intensity and peak area observed under the evaluated conditions (Figure S11). In contrast, as expected, introducing 20% glycerin into 0.5 molL^–1^ KCl caused a velocity drop of about 63% (Figure 4) compared to pure water, as seen in Supplementary Video 2. It is well-known that, for a capillary-based device driven by cellulose fibers, subtle changes in concentration affect flow properties.^39^

**Figure 4:**
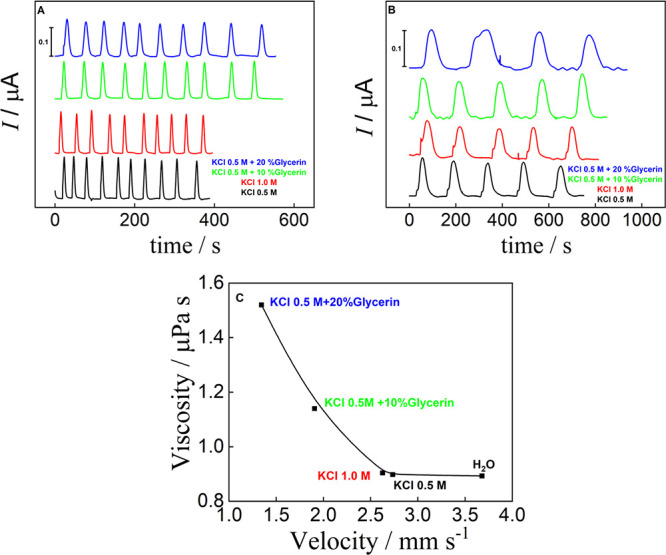
Fluid properties for different viscosities on the electrochemical response and flow velocity. (A) Transient current signals for injections of 6 μL 5 mmol L^–1^ [Fe­(CN)_6_] ^3–/4–^ in using different carrier solutions: (A) cellulose sponge and (B) Whatman 4 paper: black line: 0.5 mol L^–1^ KCl, red line: 1.0 mol L^–1^ KCl, green line: 0.5 mol L^–1^ KCl + 10% Glycerin and blue line: 0.5 mol L^–1^ KCl + 20% Glycerin. (C) Viscosity as a function of velocity obtained from dye solution using the sponge as the passive pump. Conditions: E_det_ = +0.35 V vs carbon pseudo-RE, passive pump: cellulose sponge, distance Inj-WE: 30 mm, Configuration D, DSA468. Error bars show the average and standard deviations of 10 injections.

Regarding the electrochemical signal, we observed that peak resolution is less affected than flow velocity, as the current intensity decreased by approximately 20% when comparing 0.5 mol·L^–1^ KCl with 0.5 mol·L^–1^ KCl containing 20% glycerin (see Figure S12). In the case of KCl 0.5 mol·L^–1^ combined with 20% glycerin, the peak broadened by approximately 40% compared to KCl 0.5 mol·L^–1^ with only a 16% decrease in current intensity (Figure S12). These results suggest that, despite a nearly 60% reduction in flow velocity, velocity does not significantly alter the signal, indicating that it is more dependent on the electrochemical sensor printing process. Unlike Pittman et al.,^40^ who reported that viscosity influenced the electrochemical signal, our device demonstrates that using a sponge as a passive pump effectively minimizes this effect. This achievement is significant for laminate-based microfluidic systems, as it enables reliable electrochemical analysis across a range of solution viscosities without compromising signal integrity.

Viscosity was also assessed using a fan-shaped Whatman 4 paper pump, often used as a waste pad or passive pump in laminate-based microfluidic devices.^21,30,37,41^ For this comparison, all device and measurement parameters were kept constant: the same channel geometry, inlet volume, carrier electrolyte, analyte concentration, and viscosity range were used for both pumps. The only variable was the outlet material cellulose sponge or Whatman 4 paper. We did not attempt to match the void volume or total capillary capacity of paper and sponge; thus, the comparison reflects their practical performance as off-the-shelf pumping elements rather than a fully normalized materials study. The amperometric signals, obtained using paper instead of a sponge, resulted in poor resolution peaks, particularly at higher viscosities (see Figure 4B). Additionally, the number of injections that can be performed with a single buffer loading is limited, affecting the analytical frequency compared to the sponge setup (15 and 65 injections per hour for paper and sponge, respectively). This issue was especially pronounced with solutions containing 20% glycerin, where peaks became broad and lost resolution entirely (see Figure 4B). This difference may be attributed to enhanced capillary pumping in the sponge, driven by the way these cellulose materials are arranged or integrated into the sponge matrix when compared to paper (see Figure S13 (SEM images)), which can influence fluid transport efficiency and overall absorption behavior. The SEM micrographs show that the sponge and the Whatman 4 paper have markedly different microstructures, which explain their distinct fluid-handling behaviors. The sponge, as shown in Figure S13A, exhibits a highly porous, three-dimensional architecture with large, irregular cavities connected by thin walls, resulting in a high void fraction and enabling it to absorb and store substantial liquid volume with relatively low flow resistance within the bulk. In contrast, the Whatman 4 paper (Figure S13B) presents a dense, randomly entangled network of cellulose fibers that forms a more planar sheet, with numerous smaller interfiber pores that create tortuous yet well-defined capillary pathways. This fibrous structure supports rapid wicking and through-plane flow while providing coarse particle retention, meaning the paper acts primarily as a wicking and filtering medium, whereas the sponge behaves more like a thick, compressible absorbent reservoir. These results highlight the limitations of using paper as a waste pad or pump, particularly for high-viscosity solutions, making it unsuitable for the reliable analysis of biological fluids.

Under the optimal conditions, the analytical curve for [Fe­(CN)_6_]^3–^/^4–^ was constructed in the lower and higher viscosities analyzed previously: 0.5 mol L^–1^ KCl and 0.5 mol L^–1^ KCl + Glycerin 20%, as shown in Figure S14. For both conditions, the calibration curve (Figure S14C and D) was linear from 1 to 8 mmol L^–1^, according to the equation I­(μA) = 0.01643 (±0.00153) + 0.02409 (±0.00038) C (C = concentration of [Fe­(CN)_6_]^3–^/^4–^ in mmol L^–1^), (R^2^ = 0.997) and I­(μA) = 0.01773(±0.00175) + 0.02037 (±0.00043) C (R^2^ = 0.997), using as carrier solution the 0.5 mol L^–1^ KCl and 0.5 mol L^–1^ KCl + Glycerin 20%, respectively. The analytical curve slope in the presence of Glycerin was approximately 7% lower than in its absence. This minor difference indicates that the proposed device delivers consistent analytical performance regardless of the solution’s viscosity. The limits of detection (LOD) for [Fe­(CN)_6_]^3–^/^4–^ in 0.5 mol L^–1^ KCl and 0.5 mol L^–1^ KCl + Glycerin 20% were 0.190 and 0.257 mmol L^–1^, respectively, where LOD = 3sdB/S, S corresponds to the slope, and sdB is the standard deviation of the intercept from the calibration curve. These findings suggest the strong possibility for the device’s use as a point-of-care sensor, as its performance remains stable even in more viscous media.

### Species Detection in the Viscous Solutions

To demonstrate the capability for analysis in biofluids, we evaluated the analytical performance of our device for the detection of three model species commonly found in saliva (nicotine, dopamine, and paracetamol) using a viscous solution. Analytical curves were constructed using 0.1 mol L^–1^ PBS as the carrier solution, and viscosity adjustments were made only for the standards to simulate the saliva analysis. To ensure that the standard solution was compatible with the viscosity of real human saliva, we added 0.50% (w/v) SCMC, corresponding to 1.80 mPa s, to the standard solutions.^42^

Figure 5A and B show the amperometric signals for increasing concentrations of the compounds and the corresponding analytical curves. The amperometric profiles remained consistent as expected, regardless of variations in solution viscosity, even in the absence of viscosity adjustments in the carrier solution. Also, the low standard deviation (inset figures) confirms the good reproducibility across the three different devices.

**Figure 5:**
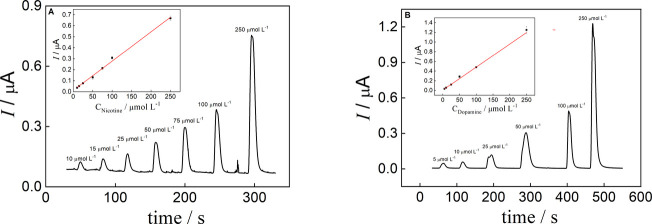
Amperometric measurements were conducted with injections of 5 μL of each analyte using 0.1 mol L^–1^ PBS as carrier solution and standards containing 0.5% SCMC. The flow velocity was calculated considering the time for a dye solution to travel through the straight channel. (A) Nicotine and (B) dopamine. (Error bars indicate the standard deviation of injections in 3 different devices). Conditions: E_Nicotine_ = +0.90 V and E_Dopamine_ = 0.25 vs Ag/AgCl, passive pump: sponge, distance Inj-WE: 30 mm, Configuration D, DSA468.

Table S4 shows the analytical parameters reached for nicotine and dopamine. The analytical parameters for dopamine and nicotine were similar to those reported in the literature for carbon-based electrodes, with LODs of 3 μmol L^–1^ ^43,44^ and 9 μmol L^–1^,^45–47^ respectively. These results demonstrate that the proposed microfluidic platform, integrating a laminate capillary-driven microfluidic device with flow regulation mediated by a commercial sponge, presents robust analytical performance in the analysis of viscous samples.

The consistent amperometric responses, despite variations in fluid viscosity, highlight the capability of the device to handle complex matrices without requiring viscosity normalization. This characteristic is particularly advantageous for point-of-care (POC) diagnostics, where sample pretreatment, mainly the viscosity adjustment, must be minimal or absent. The ability to reliably analyze biofluids such as saliva, which exhibit natural variability in viscosity, underscores the potential of this system as a versatile and user-friendly POC sensor. These findings open the perspectives for further development of autonomous (ELISA-type) diagnostic devices suitable for real-world clinical and field applications.

### Generator-Collector Mode for Paracetamol Sensing in Human Saliva

Paracetamol (PAR) is an analgesic medication commonly used to alleviate symptoms of fever and provide pain relief. While it is beneficial when taken in therapeutic doses, excessive use can pose serious risks, including severe liver and kidney damage. Both intentional and unintentional overdoses contribute significantly to the growing number of emergency hospital admissions, a trend that has become increasingly prevalent in recent years.^48^ Among these, the misuse of PAR is of particular concern. Due to its widespread availability and perceived safety, paracetamol-related overdoses have emerged as a significant public health issue.^49^ For example, the American Association of Poison Control Centers reported over 80,000 cases of acetaminophen overdose in 2021 alone.^50^ Thus, early and rapid diagnosis of PAR overdose is crucial for initiating appropriate treatment and preventing serious health complications. Therefore, developing analytical methods to quantify PAR is a helpful strategy for monitoring PAR levels in biological fluids.

For the PAR quantification, we applied the generator-collector mode (GC). The product formed during the electrochemical oxidation of PAR at +0.44 V, N-acetyl-p-benzoquinoneimine (+0.01 V), can be electrochemically reduced and used for its indirect detection under these conditions (Figure S15). This approach not only facilitates the detection of PAR with minimal interference at a detection potential (−0.05 V corresponding to the reduction process), but it also highlights the versatility of the proposed device. The device is designed to integrate an additional working electrode for sample pretreatment on a single platform.

Figure 6A presents a schematic of the electrode array used for electrochemical measurements, along with the corresponding amperometric signals. The current response of each electrode was monitored simultaneously using amperometry. A potential of 0.9 V vs. Ag/AgCl was applied to the generator electrode, while – 0.05 V vs Ag/AgCl was applied to the collector. Two transient current curves were obtained: a positive current indicating the oxidation of both the saliva matrix and the generation of paracetamol-reduced species and a negative current corresponding to the subsequent reduction of paracetamol at the collector electrode, as shown in Figure 6B. Calibration curves were constructed using the proposed GC mode in the linear range from 10 to 300 μmol L^–1^ PAR (Figure 6C,D), according to the following equations: Ip = 0.05105 (±0.01470) + 0.01836 (±0.00005)­C_PARgen_ and Ip = 0.00679 (±0.00181) + 0.0294 (±0.00005)­C_PARcol_, for the generator and collector electrodes, respectively. From these, the analytical frequency was estimated to be 45 injections per hour.

**Figure 6:**
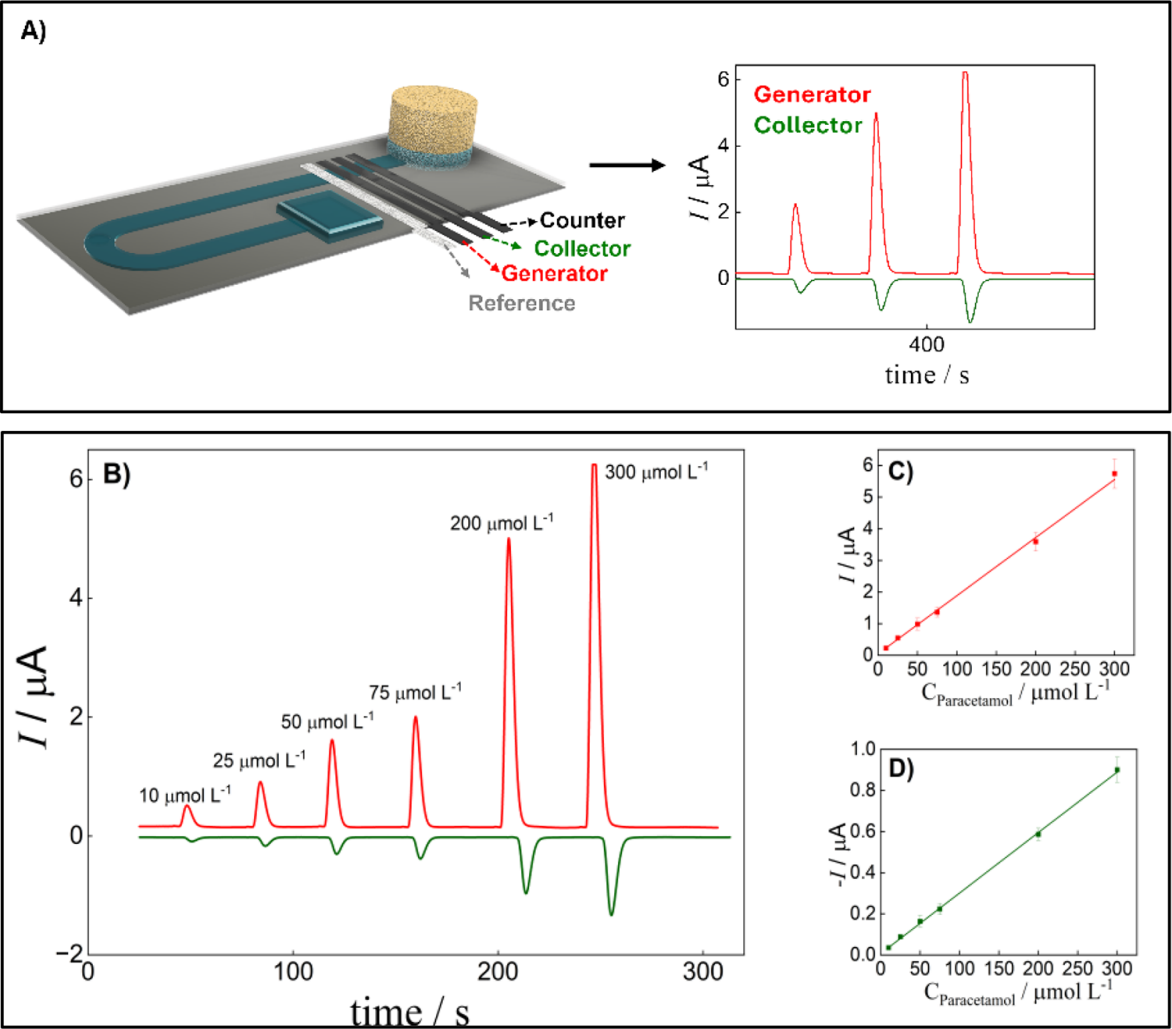
Evaluation of GC mode to quantify the PAR. (A) Schematic representation of the GC microfluidic device used and the representative amperometric graph obtained under these conditions. (B) Transient current signals for injections of 4 μL of different PAR concentrations in 0.5% SCMC at the generator electrode (red line) and collector electrode (green line). Calibration curve obtained for PAR: (C) generator and (D) collector electrodes (error bars indicate the standard deviation of injections in three different devices). Conditions: E_generator_ = +0.90 V and E_collector_ = −0.05 vs Ag/AgCl, passive pump: sponge, distance Inj-WE: 30 mm, Configuration D, DSA468.

LODs were estimated to be 2.0 and 1.8 μmol L^–1^ for the generator and collector electrodes, respectively. It is important to highlight that the achieved LOD is enough for monitoring PAR overdose cases, as concentrations in such instances typically exceed 65 μmol L^–1^.^51^ These results suggest that even though the signal was lower for the collector electrode, this mode did not affect the sensitivity and detectability of the system.

For PAR quantification in human saliva, which evaluates the sponge microfluidic device’s ability to handle real samples, 4 μL of undiluted saliva from anonymous donors were directly applied to the sample injection without any sample preparation. To evaluate the accuracy of the proposed method, the analyzed samples were subjected to recovery experiments by spiking them with known concentrations of PAR before analysis. Figure S16 shows the amperometric signal recorded for human saliva and for human saliva spiked with PAR. All quantifications were performed in triplicate, and the PAR contents were determined using an equation obtained from an external analytical curve in collector mode constructed with SCMC at 0.5% to achieve the desired viscosity (Table 1).

For unspiked samples, a higher signal (see Figure S16) was observed in generator mode, corresponding to the oxidation of matrix compounds. In contrast, no amperometric signal was detected in collector mode, indicating the absence of paracetamol. These findings suggest that, under oxidation conditions, numerous electroactive species in the matrix may interfere with the PAR signal. However, in PAR-spiked saliva samples, a distinct cathodic current was recorded in collector mode, confirming the formation of the reduced PAR product. The recovery results for both samples (Table 2) indicate that recovery percentages of nearly 100% were achieved, regardless of the PAR concentration added. This is good evidence that other species from the sample matrix did not interfere in the amperometric determination of PAR in collector mode.

**Table 2:** Analytical results for PAR determination in human saliva samples

human saliva	PAR added (μmol L^–1^)	PAR found (μmol L^–1^)	recovery (%)	RSD^a^ (%)
1	20	18.3 ± 1.9	91 ± 10	10
	50	46.6 ± 2.6	93 ± 6	6
	150	127.9 ± 8.1	85 ± 6	7
2	20	16.9 ± 2.4	84 ± 12	14
	50	55.5 ± 5.4	111 ± 11	10
	150	151.9 ± 9.6	101 ± 7	7
3	20	18.3 ± 0.9	91 ± 5	8
	50	53.2 ± 8.6	106 ± 17	16
	150	138.5 ± 15.5	92 ± 10	11

a Relative standard deviation obtained for the three different assembled devices.

Despite the good accuracy demonstrated by the real samples analysis, the selective study was performed using the main interferent species present in saliva:^52,53^ uric acid, ascorbic acid, oxilac acid, and glucose. As shown in Figure S17, the species analyzed did not significantly interfere since they changed the voltammetric signal of PAR by <10%. Therefore, this demonstrates that the method is capable of selectively detecting PAR even in complex biological matrices containing multiple interfering substances. The results also indicate that the sensor remained free from fouling despite the high protein content present in human saliva.

## Conclusions

Herein, analytical devices were developed using low-cost materials, with transparency and a double-sided adhesive. A commercial sponge was used to provide improved flow control compared with the paper typically applied as a waste pad in microfluidic devices. In addition, the sponge enabled a higher analytical frequency for viscous solutions when compared to paper, achieving 15 and 65 injections per hour for paper and sponge, respectively. Beyond providing effective flow control, the device’s design enables flow modification by replacing the passive pump sponge. The screen-printed method was used to create the electrochemical detector on this device. This work systematically optimized the fabricating method, demonstrating that the response is similar regardless of the type of material used, indicating that the material used to make the capillary-driven device does not significantly affect the flow velocity. Analytical calibration curves for nicotine and dopamine were constructed in a viscous solution, reaching the LOD comparable to other reports, demonstrating the feasibility of analyzing these compounds in viscous biological fluids. Also, the sponge device was used to quantify paracetamol in human saliva without requiring any dilution. It is important to note that a key finding presented in this project was the ability to analyze viscous solutions, such as biological fluids, without compromising the electrochemical signal, even when the flow rate was altered. This method provides a wide forms to develop the device, such as the two working electrode systems with the generator-collector settings. Also, the new design using the sponge as a passive pump opens the possibility of making the reactions (sample preparation steps) on the microfluidic channel, since the flow could be controlled by simply replacing the passive pump. Therefore, these results pave the way for the development of capillary-driven microfluidic devices with electrochemical detection for saliva analysis outside laboratory settings.

## Supplementary Material







## Notes

Schematic representation of microfluidic device, voltametric responses of screen-printed for [Fe­(CN)_6_]^3–/4–^, evaluation of the electrodes positions, evaluation of the distance injection to detector, evaluation of channel height, contact angle of transparency, evaluations of configurations arrays, schematic representation of the device with 500 μL, evaluation of the inlet volume, evaluation of the viscosities solution, evaluation of the viscosities solution using the paper as waste pad, analytical curve for [Fe­(CN)_6_]^3–/4–^ in different viscosities, analytical parameters obtained from dopamine and nicotine, voltametric responses of screen-printed for paracetamol, amperometric response for saliva analysis, interference study. (PDF)
Measurement in the proposed device (MP4)
Introducing 20% glycerin into 0.5 mol·L^–1^ KCl, causing a velocity drop of about 63% compared to pure water (MP4)
