# Supplementary material for: Sponge-Based Flow Control in Laminate Capillary-Driven Electrochemical Microfluidic Devices for Viscous Sample Analysis
Source: Anal Chem. 2026 Mar 30;98(14):10521–31. doi: 10.1021/acs.analchem.5c07262 (PMC13084631; doi:10.1021/acs.analchem.5c07262)
Supplement: Supplementary file 1 [file ac5c07262_si_001.pdf]

Supplementary Material

Sponge-Based Flow Control in Laminate Capillary-Driven Electrochemical Microfluidic Devices for Viscous Sample Analysis

Diele. A. G. Araújo[a], Thaisa A. Baldo[b], Thiago R. L. C. Paixão[a] and Charles S. Henry[b,c]*

[a] Institute of Chemistry, Department of Fundamental Chemistry, University of São Paulo, 05508-000, São Paulo-SP, Brazil.

[b] Department of Chemistry, Colorado State University, Fort Collins, CO, 80523, USA.

[c] School of Biomedical and Chemical Engineering, Colorado State University, Fort Collins, CO, 80523, USA.

KEYWORDS: screen-printed electrode, biological fluids, human saliva analysis, paracetamol, generation-collection system.

Table S1: Comparison between the paper and the sponge as a passive pump......................4

Figure S1. Schematic representation of the curve design coupled to the generator-collector system to quantify paracetamol in saliva. 4

Figure S2. Flow velocity in function of the successive injections obtained using the straight channel coupled with different sponge materials as passive pumps. Flow velocity was calculated based on the time it took for 4 μL of dye solution to travel through the straight channel. 5

Figure S3. Cyclic voltammograms recorded in static mode using a screen-printed electrode in 0.5 mol L^{-1} KCl in the presence of 5 mmol L^{-1} $[Fe(CN)_6]^{3-/4-}$; ν = 50 mV s^{-1}. 6

Figure S4. Transient current signals for injections of 5 μL 5 mmol L-1 $[Fe(CN)_6]^{3-/4-}$ in 0.5 mol L^{-1} KCl using the different configurations as schematic representations: **A)**1; **B)** 2; **C)** 3. **D)** Ip and area as a function of the different electrode positions (Error bars indicate the standard deviation of I_p and area for 7 successive injections). Carrier solution: 0.5 mol L^{-1} KCl. E_{det} = +0.35 V vs. carbon pseudo-RE, passive pump: cellulose sponge, distance Inj-WE: 10 mm. 7

Figure S5. Transient current signals for injections of 5 μL 5 mmol L-1 [Fe(CN)6]3−/4− in 0.5 mol L−1 KCl in the different distance injection samples to working electrode: A) 10; B) 20; C) 30 and D) 40 mm. E) Ip and area as a function of the successive injections (Error bars indicate the standard deviation of Ip for 10 successive injections). Carrier solution: 0.5 mol L−1 KCl. Edet = +0.35 V vs. carbon pseudo-RE, passive pump: cellulose sponge..............8

Figure S6. I_p and area as a function of the different straight channel heights. (Error bars indicate the standard deviation of Ip for 10 successive injections). Carrier solution: 0.5 mol L^{-1} KCl. Edet = +0.35 V vs. carbon pseudo-RE, passive pump: cellulose sponge, distance Inj-WE: 30 mm. 9

Figure S7. Contact angle determination by dropping 4 μL of water on different transparencies was evaluated. 10

Figure S8. Ip and area as a function of the different arrays using the two transparency types. (Error bars indicate the standard deviation of Ip for 10 successive injections). Carrier solution: 0.5 mol L−1 KCl. Edet = +0.35 V vs. carbon pseudo-RE, passive pump: cellulose sponge, distance Inj-WE: 30 mm, DSA468..11

Figure S9. Schematic representation of the proposed device with 500 μL inlet. 12

Figure S10. Ip and area as a function of using the different inlet volumes. (Error bars indicate the standard deviation of Ip for 10 successive injections). Carrier solution: 0.5 mol L−1 KCl. Edet = +0.35 V vs. carbon pseudo-RE, passive pump: cellulose sponge, distance Inj-WE: 30 mm 13

Table S2. Optimization conditions evaluated and selected in the device fabrication. 13

Table S3. Viscosity of the solution studied.[4, 5] 13

Figure S11. I_p and peak area as a function of carrier solutions evaluated (Error bars indicate the standard deviation of 10 successive injections). Conditions: E_{det} = +0.35 V vs. carbon

pseudo-RE, passive pump: cellulose sponge, distance Inj-WE: 30 mm, Configuration D, DSA468..........14

Figure S12. Influence of the solution viscosities on the electrochemical response using a paper as a waste pad/passive pump. Ip and peak area as a function of carrier solutions evaluated (Error bars indicate the standard deviation of 10 successive injections). Conditions: Edet = +0.35 V vs. carbon pseudo-RE, passive pump: Whatman 4, distance Inj-WE: 30 mm, Configuration D, DSA468. 15

Figure S13: SEM micrographs of a **(A)** Sponge and **(B)** Whatman 4 paper. Micrographs taken with a magnification of x75. Morphological properties were examined by scanning electron microscopy (SEM). These measurements were carried out using a JEOL JSM-IT800 (HL) microscope supplied by JEOL Microscopes (Waltham, MA, USA). 16

Figure S14: Influence of the KCl viscosities on the electrochemical signal of $[Fe(CN)_6]^{3-/4-}$. Transient current signals for injections of 6 µL in different $[Fe(CN)_6]^{3-/4-}$ concentrations using 0.1 mol L^{-1} KCl as carrier solution in different viscosities and respective analytical curve. **A and B)** Glycerin 0% and **C and D)** Glycerin 20%. (Error bars indicate the standard deviation of injections in 3 devices). Conditions: $E_{Nicotine}$ = +0.90 V and $E_{Dopamine}$ = 0.25 vs. Ag/AgCl, passive pump: sponge, distance Inj-WE: 30 mm, Configuration D, DSA468.....17

Table S4. Analytical parameters obtained from dopamine and nicotine. 17

Figure S15. Cyclic voltammograms recorded in static mode using a screen-printed electrode in 0.01 mol L−1 PBS buffer at pH 7.0 in the presence of 100 µmol L−1 PAR; ν = 50 mV s−1. 18

Figure S16. Transient current signals for injections of 4 µL of the real human saliva. A) unspiked in the generator (yellow line) and collector (red line) and B) spiked with PAR 20 (black line), 50 (red line) and 150 µmol L−1 (blue line) at the GC mode. Conditions: Egenerator = +0.90 V and Ecollector = -0.05 vs. Ag/AgCl, passive pump: sponge, distance Inj-WE: 30 mm, Configuration D, DSA468. 19

Figure S17. Effect of possible interfering species on the voltametric signal of BPA, presence of 100 µmol L−1 PAR: oxalic acid 200 µmol L−1, uric acid 100 µmol L−1, glucose 500 µmol L−1 and ascorbic acid 200 µmol L−1. Conditions: Egenerator = +0.90 V and Ecollector = -0.05 vs. Ag/AgCl, passive pump: sponge, distance Inj-WE: 30 mm, Configuration D, DSA468..........20

Table S1: Comparison between the paper and the sponge as a passive pump.

Pump material	Flow velocity (mm s^{-1})	Ref.
Whatman 1 paper	0.46	[1]
Whatman 1 paper	0.9	[2]
Whatman 4 paper	2.7	[3]
Cellulose sponge	4.61	**This work**
Fiber/resin sponge	1.28	**This work**

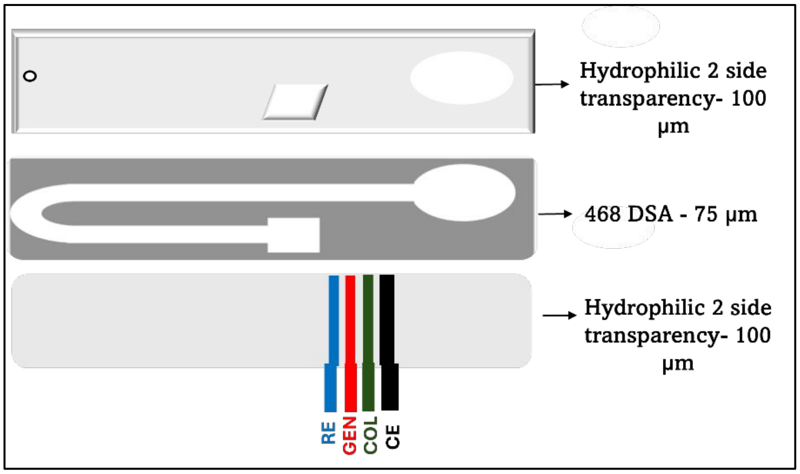

Figure S1. Schematic representation of the curve design coupled to the generator-collector system to quantify paracetamol in saliva.

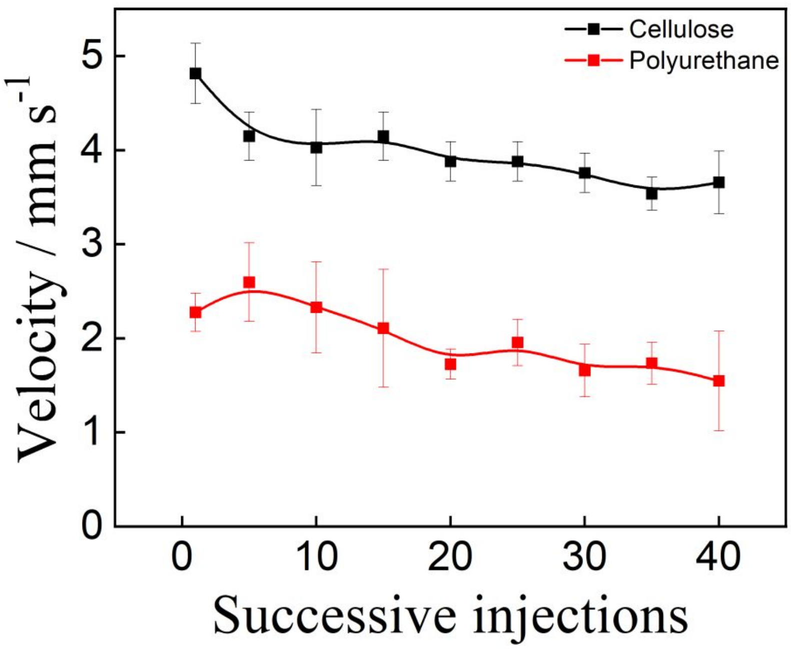

Figure S2. Flow velocity in function of the successive injections obtained using the straight channel coupled with different sponge materials as passive pumps. Flow velocity was calculated based on the time it took for 4 µL of dye solution to travel through the straight channel.

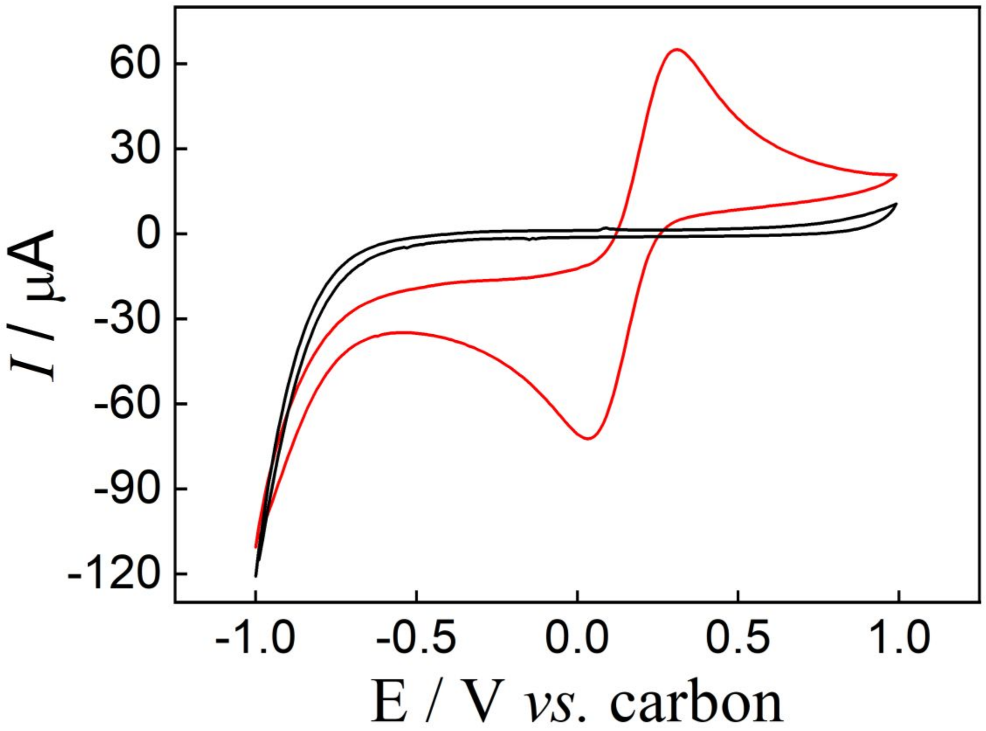

Figure S3. Cyclic voltammograms recorded in static mode using a screen-printed electrode in 0.5 mol L^{-1} KCl in the presence of 5 mmol L^{-1} $[Fe(CN)_6]^{3-/4-}$; ν = 50 mV s^{-1}.

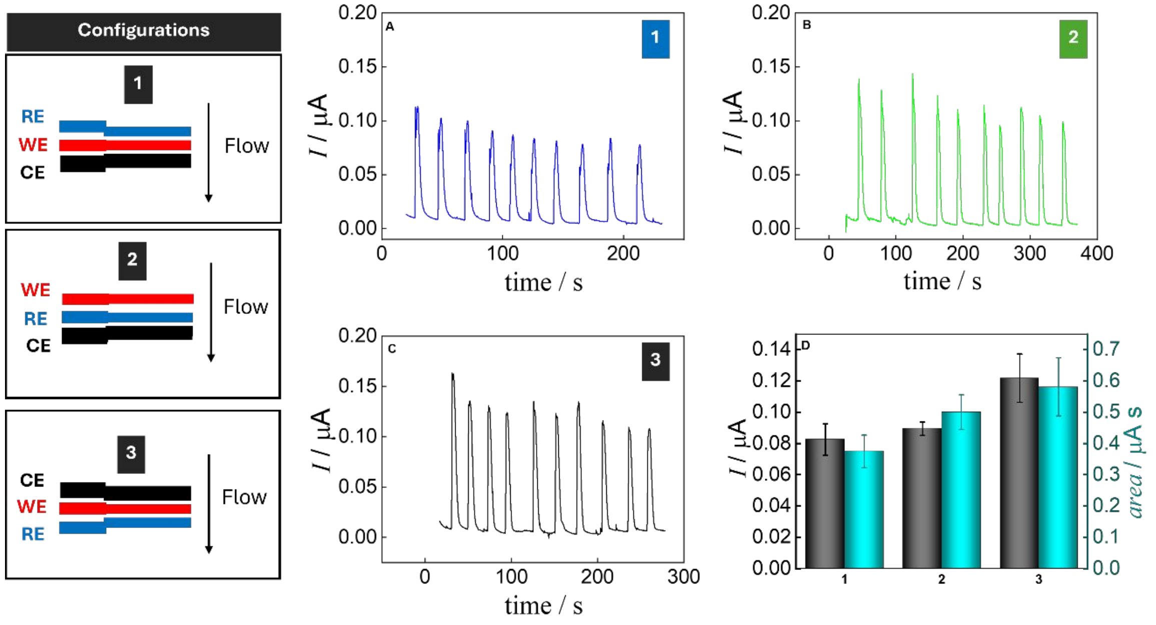

Figure S4. Transient current signals for injections of 5 µL 5 mmol L-1 $[Fe(CN)_6]^{3-/4-}$ in 0.5 mol L^{-1} KCl using the different configurations as schematic representations: **A)**1; **B)** 2; **C)** 3. **D)** Ip and area as a function of the different electrode positions (Error bars indicate the standard deviation of I_p and area for 7 successive injections). Carrier solution: 0.5 mol L^{-1} KCl. E_{det} = +0.35 V vs. carbon pseudo-RE, passive pump: cellulose sponge, distance Inj-WE: 10 mm.

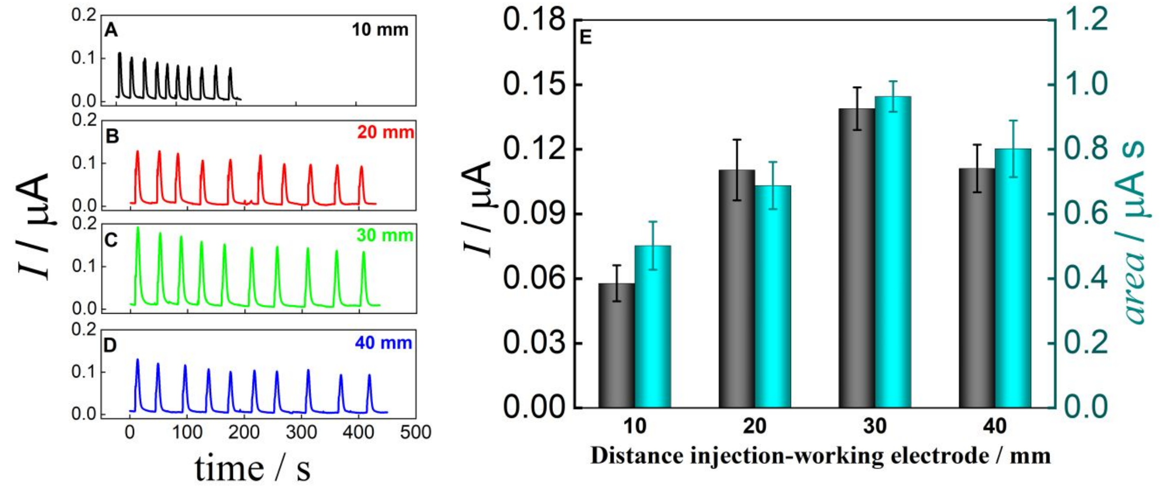

Figure S5. Transient current signals for injections of 5 μL 5 mmol L-1 $[Fe(CN)_6]^{3-/4-}$ in 0.5 mol L^{-1} KCl in the different distance injection samples to working electrode: A) 10; B) 20; C) 30 and D) 40 mm. E) Ip and area as a function of the successive injections (Error bars indicate the standard deviation of Ip for 10 successive injections). Carrier solution: 0.5 mol L^{-1} KCl. Edet = +0.35 V vs. carbon pseudo-RE, passive pump: cellulose sponge.

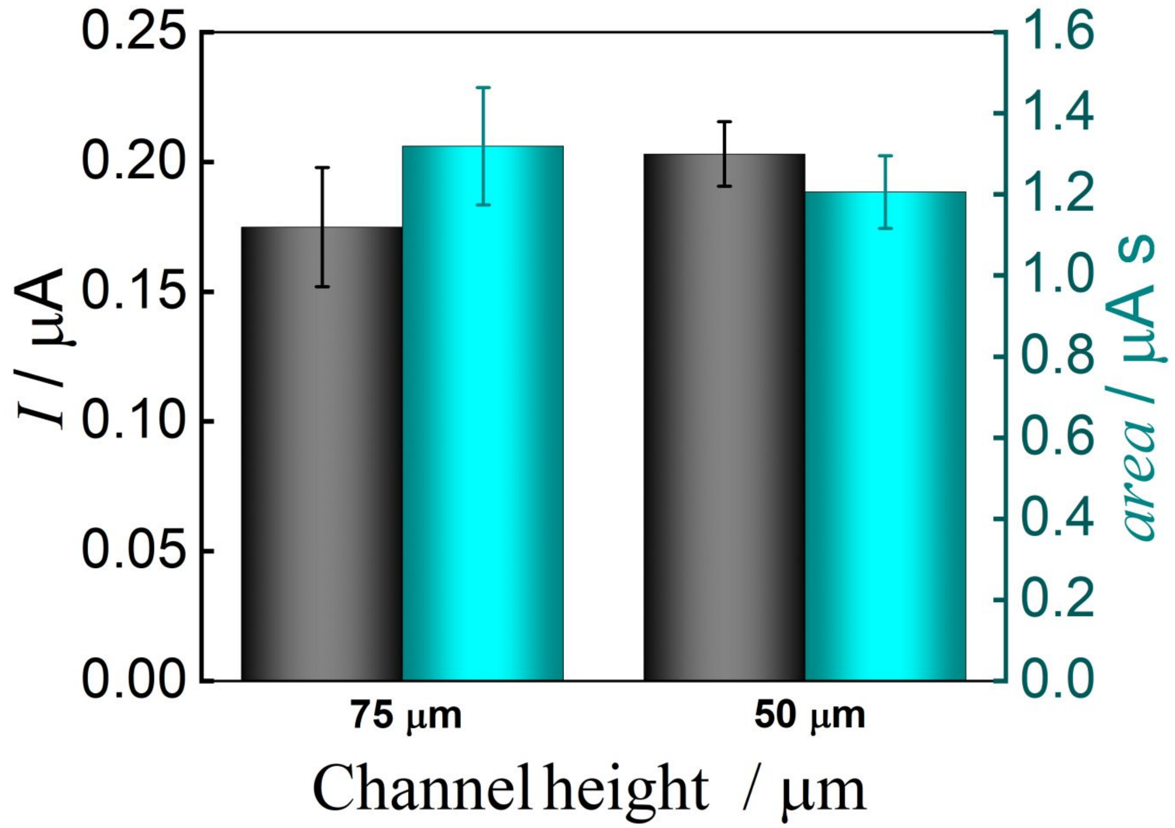

Figure S6. I_p and area as a function of the different straight channel heights. (Error bars indicate the standard deviation of Ip for 10 successive injections). Carrier solution: 0.5 mol L^{-1} KCl. Edet = +0.35 V vs. carbon pseudo-RE, passive pump: cellulose sponge, distance Inj-WE: 30 mm.

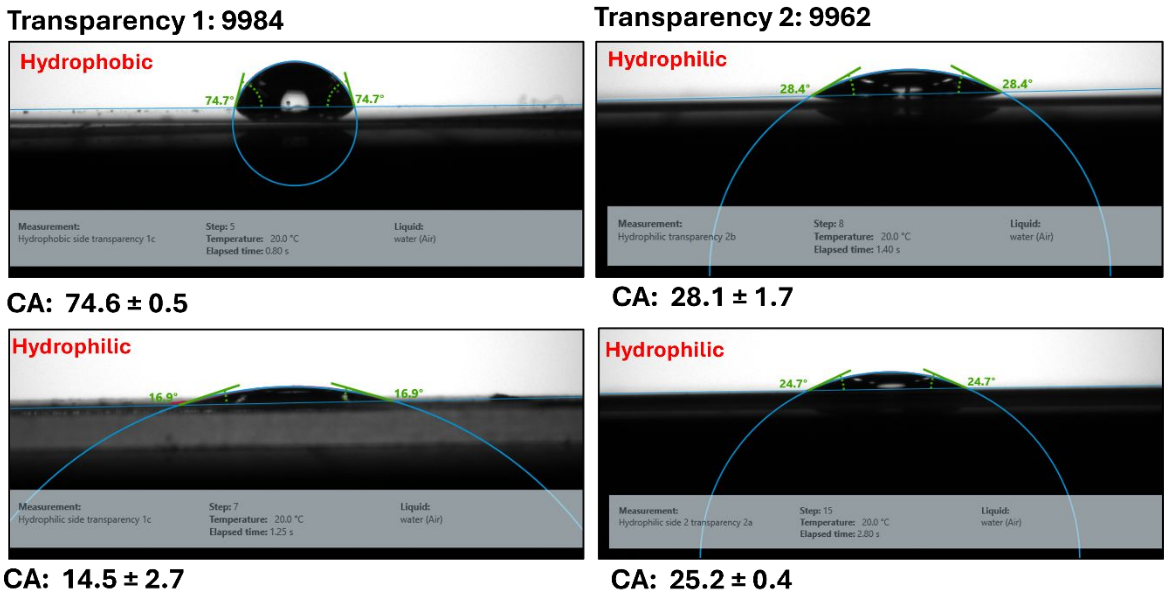

Figure S7. Contact angle determination by dropping 4 μL of water on different transparencies was evaluated.

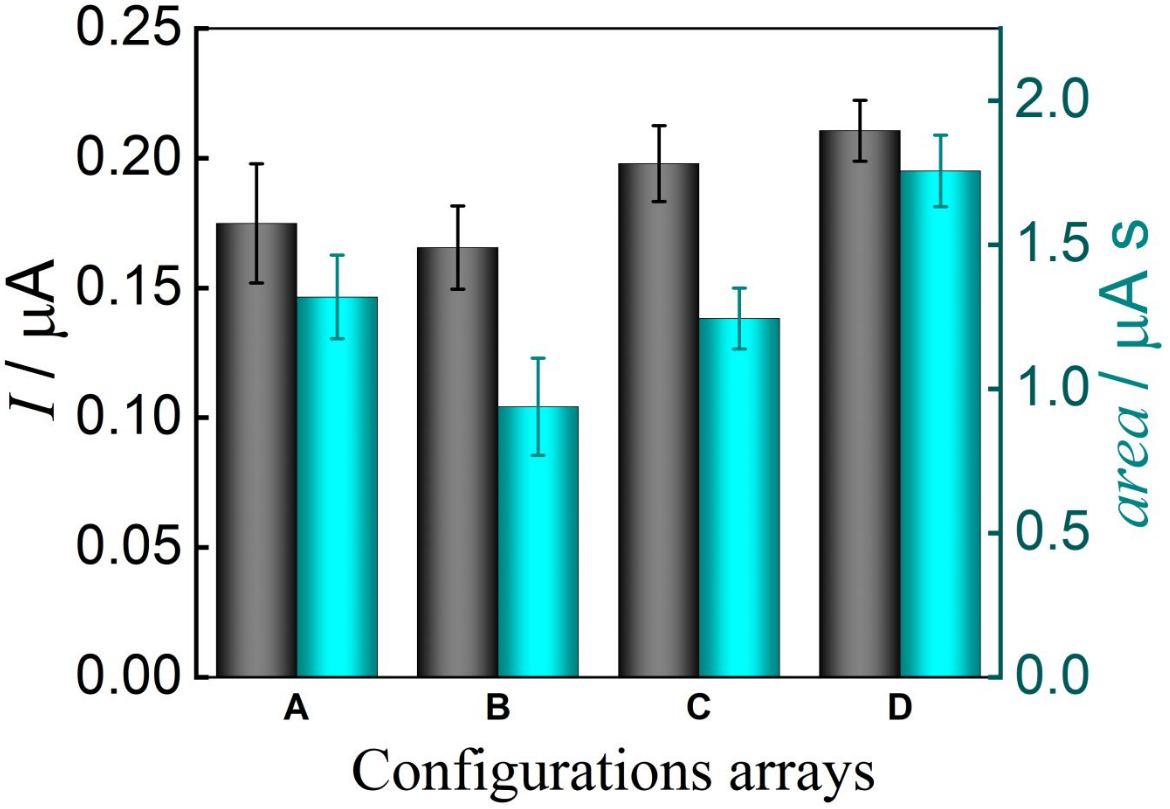

Figure S8. Ip and area as a function of the different arrays using the two transparency types. (Error bars indicate the standard deviation of Ip for 10 successive injections). Carrier solution: 0.5 mol L−1 KCl. Edet = +0.35 V vs. carbon pseudo-RE, passive pump: cellulose sponge, distance Inj-WE: 30 mm, DSA468.

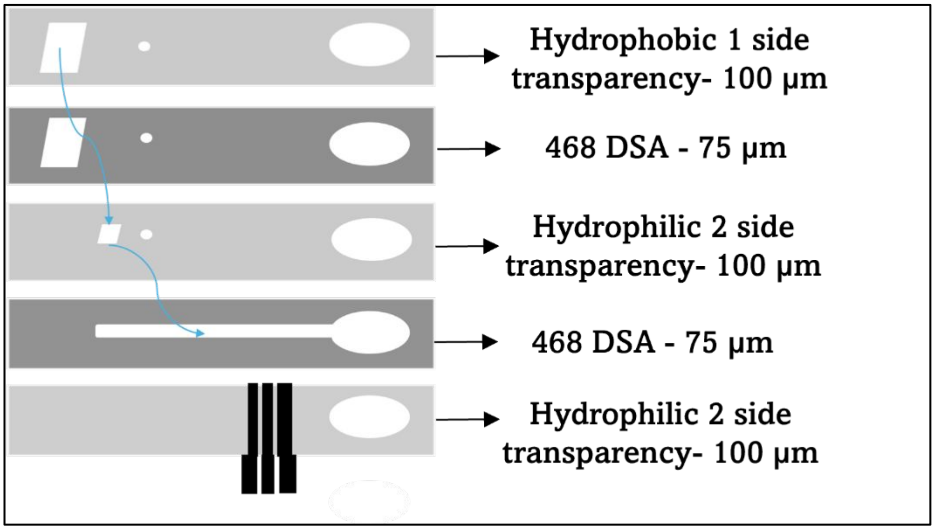

Figure S9. Schematic representation of the proposed device with 500 µL inlet.

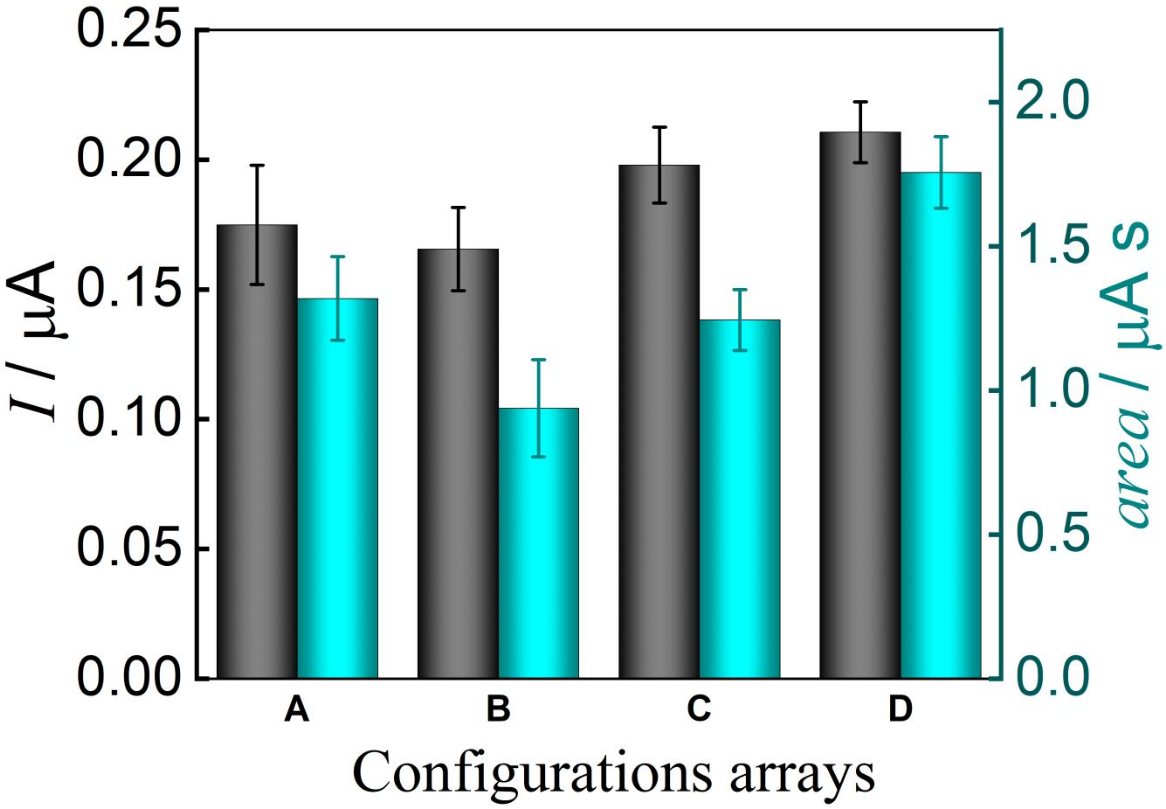

Figure S10. Ip and area as a function of using the different inlet volumes. (Error bars indicate the standard deviation of Ip for 10 successive injections). Carrier solution: 0.5 mol L−1 KCl. Edet = +0.35 V vs. carbon pseudo-RE, passive pump: cellulose sponge, distance Inj-WE: 30 mm

Table S2. Optimization conditions evaluated and selected in the device fabrication.

Conditions evaluated	Interval/types	Conditions selected
**Reference position**	First, middle and last (see Figure S3)	First
**Transparency**	Type 1 (Hydrophobic/Hydrophilic) and Type 2 (Hydrophilic)	Type 2 (Hydrophilic)
**Distance injection-working electrode**	10, 20, 30 and 40 mm	30 mm
**Channel height**	50 and 75 µm	75 µm
**Inlet volume**	200 and 500 µL	200 µL

Table S3. Viscosity of the solution studied.[4, 5]

Solution	Viscosity (µPa s)
**H_2O**	0.893
**KCl 0.5 M**	0.898
**KCl 1.0 M**	0.904
**KCl 0.5 M + 10% Glycerin**	1.14
**KCl 0.5 M + 20% Glycerin**	1.52

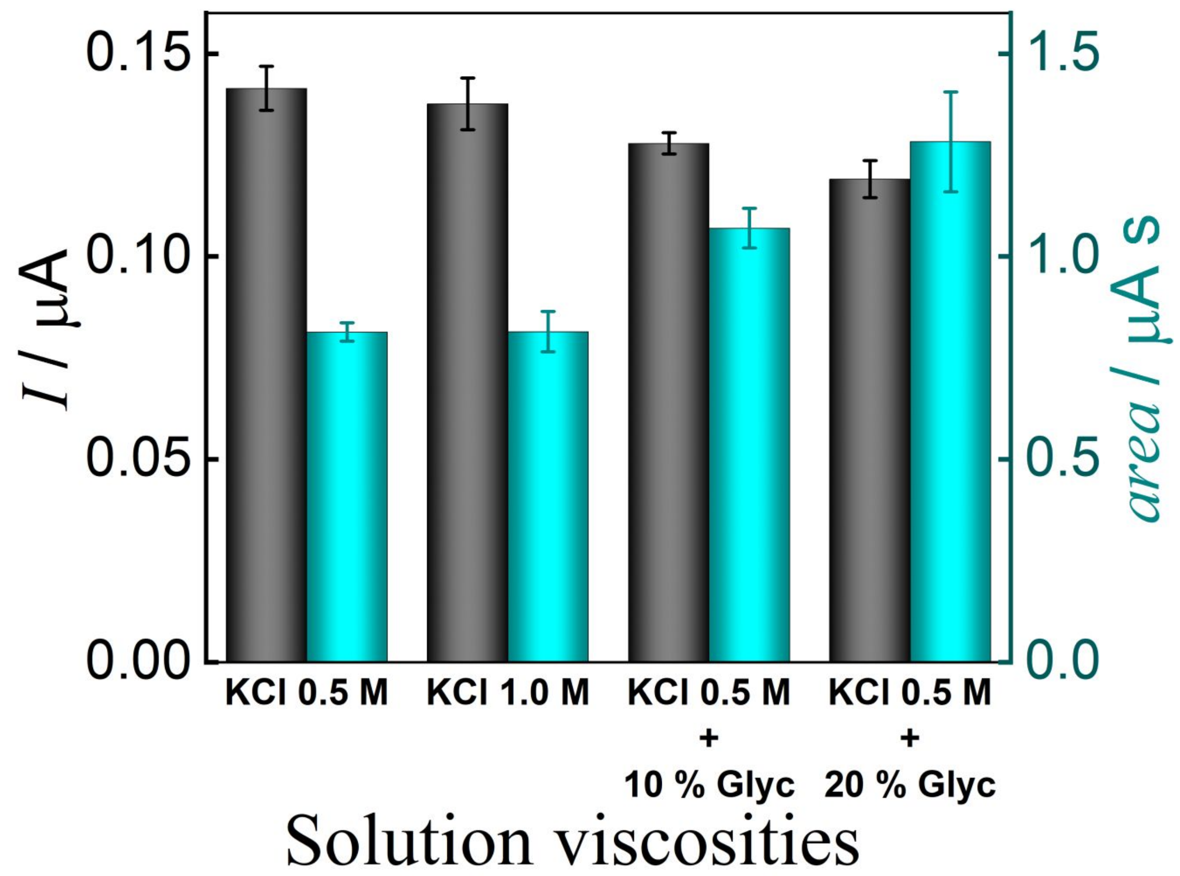

Figure S11. I_p and peak area as a function of carrier solutions evaluated (Error bars indicate the standard deviation of 10 successive injections). Conditions: E_{det} = +0.35 V vs. carbon pseudo-RE, passive pump: cellulose sponge, distance Inj-WE: 30 mm, Configuration D, DSA468.

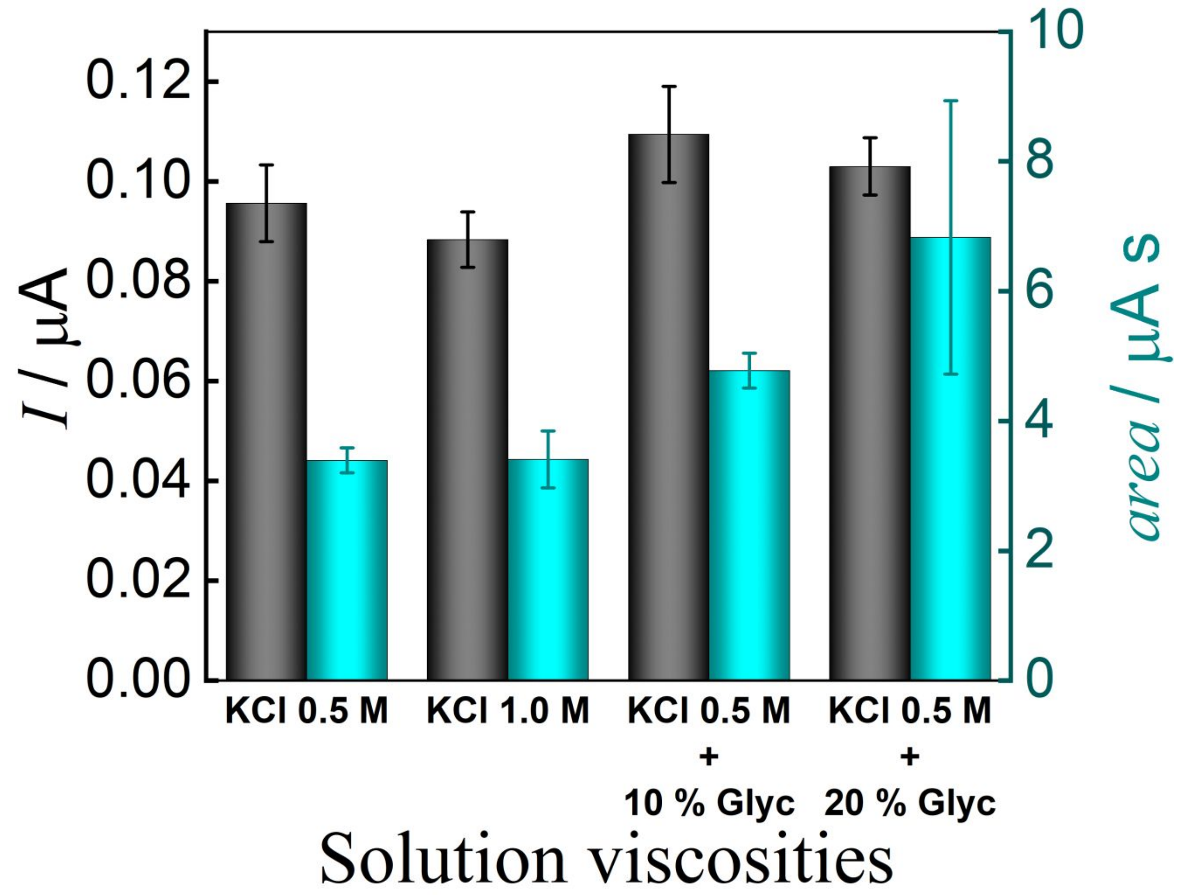

Figure S12. Influence of the solution viscosities on the electrochemical response using a paper as a waste pad/passive pump. Ip and peak area as a function of carrier solutions evaluated (Error bars indicate the standard deviation of 10 successive injections). Conditions: Edet = +0.35 V vs. carbon pseudo-RE, passive pump: Whatman 4, distance Inj-WE: 30 mm, Configuration D, DSA468.

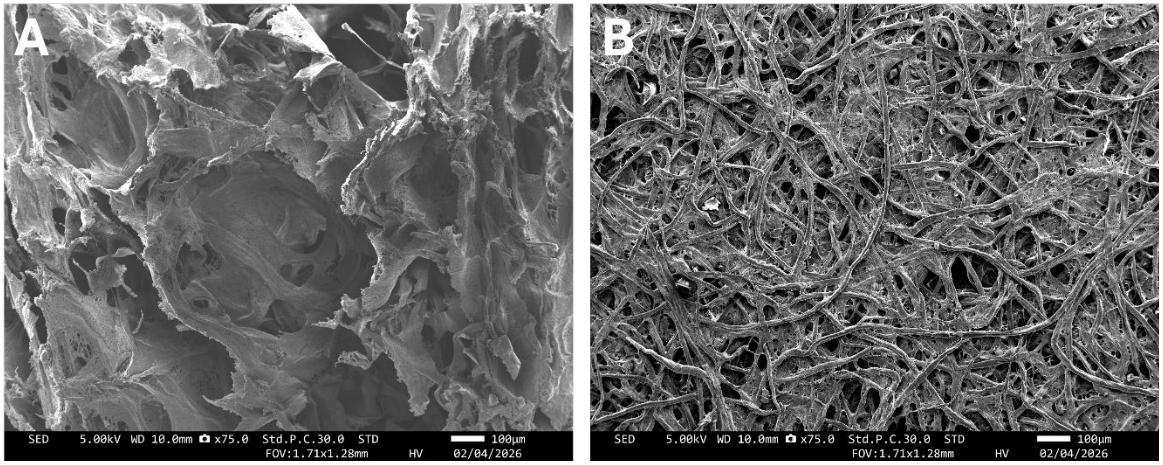

Figure S13: SEM micrographs of a **(A)** Sponge and **(B)** Whatman 4 paper. Micrographs taken with a magnification of x75. Morphological properties were examined by scanning electron microscopy (SEM). These measurements were carried out using a JEOL JSM-IT800 (HL) microscope supplied by JEOL Microscopes (Waltham, MA, USA).

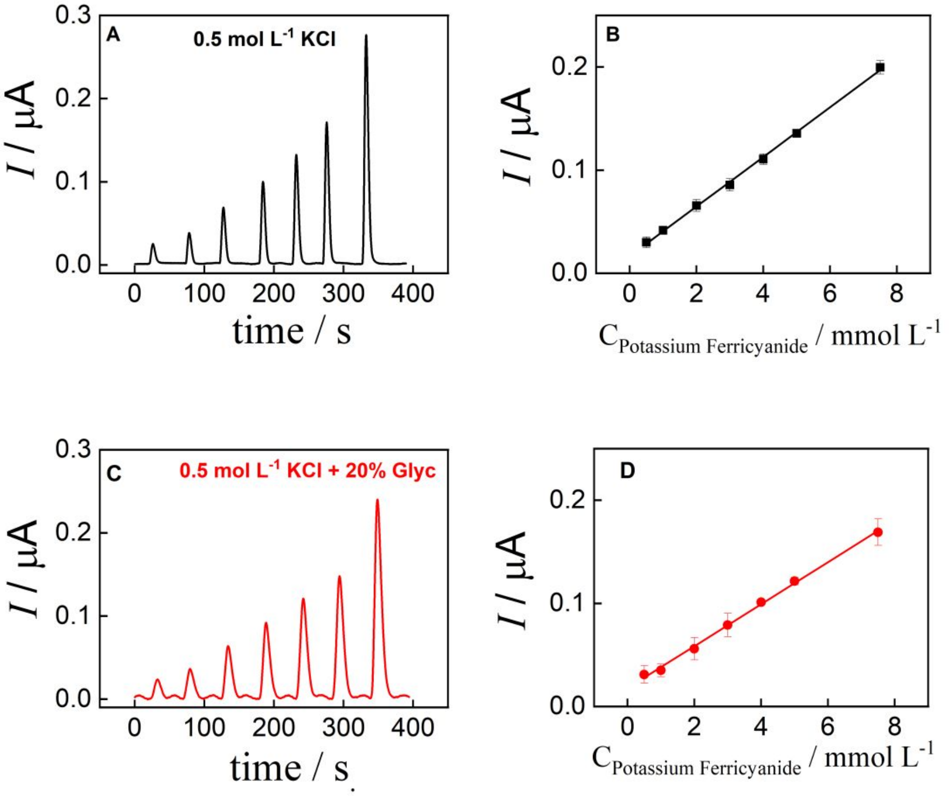

Figure S14: Influence of the KCl viscosities on the electrochemical signal of $[Fe(CN)_6]^{3-/4-}$. Transient current signals for injections of 6 µL in different $[Fe(CN)_6]^{3-/4-}$ concentrations using 0.1 mol L^{-1} KCl as carrier solution in different viscosities and respective analytical curve. **A and B)** Glycerin 0% and **C and D)** Glycerin 20%. (Error bars indicate the standard deviation of injections in 3 devices). Conditions: $E_{Nicotine}$ = +0.90 V and $E_{Dopamine}$ = 0.25 vs. Ag/AgCl, passive pump: sponge, distance Inj-WE: 30 mm, Configuration D, DSA468.

Table S4. Analytical parameters obtained from dopamine and nicotine.

Analyte	PBS 0.1 mol L^{-1} + 0.5% SCMC			
	Intercept ± SD	Slope ± SD µA µmol^{-1} L	LOD µmol L^{-1}	R^2
**Dopamine**	0.00838 ± 0.00412	0.00654 ± 0.00017	3	0.99664
**Nicotine**	0.01266± 0.00826	0.00226 ± 0.00007	9	0.99504

*Standard deviation obtained for the 10th injection in three different assembled devices.

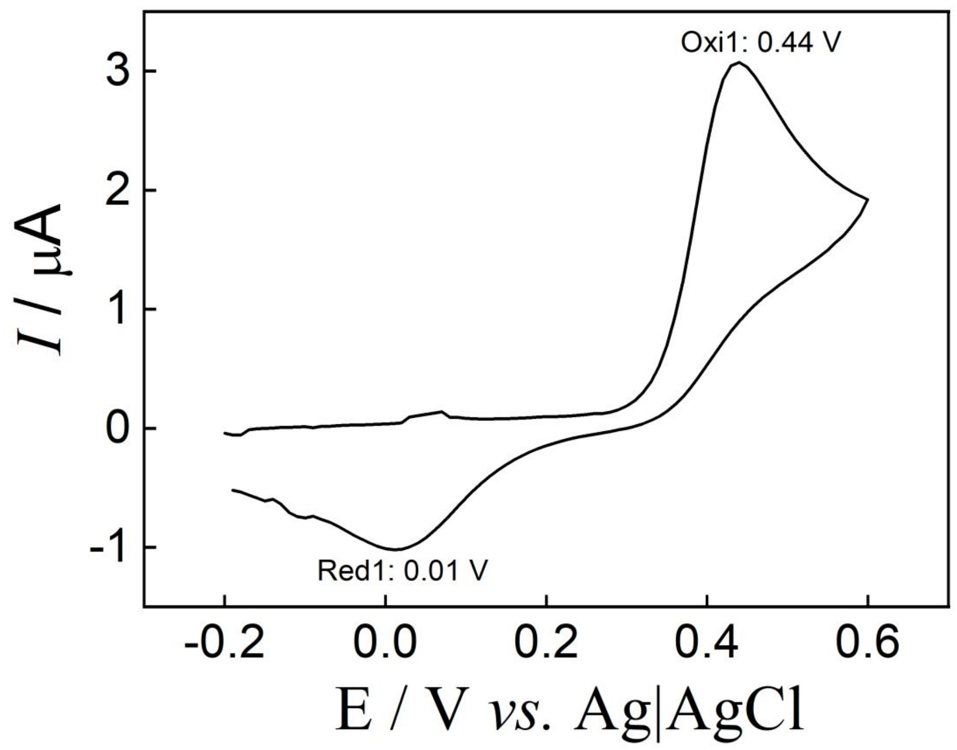

Figure S15. Cyclic voltammograms recorded in static mode using a screen-printed electrode in 0.01 mol L−1 PBS buffer at pH 7.0 in the presence of 100 μmol L−1 PAR; ν = 50 mV s−1.

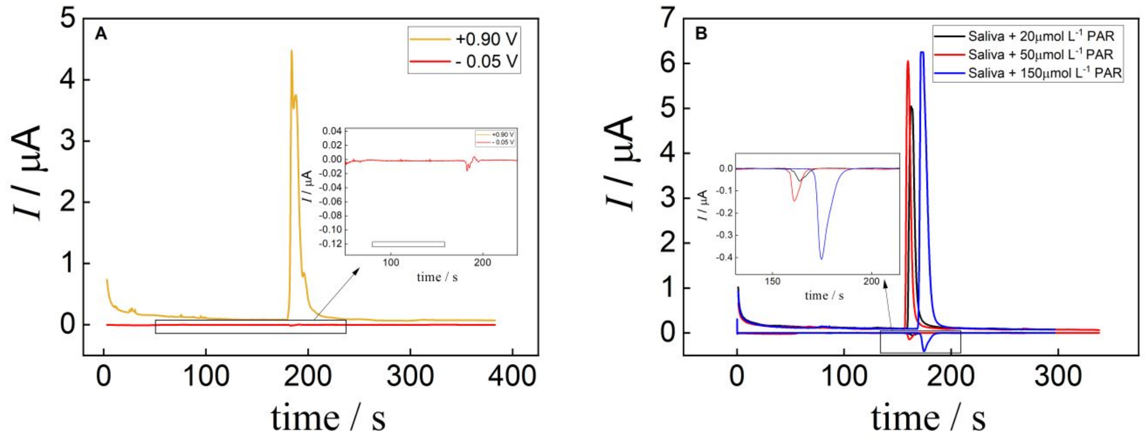

Figure S16. Transient current signals for injections of 4 µL of the real human saliva. A) unspiked in the generator (yellow line) and collector (red line) and B) spiked with PAR 20 (black line), 50 (red line) and 150 µmol L−1 (blue line) at the GC mode. Conditions: Egenerator = +0.90 V and Ecollector = -0.05 vs. Ag/AgCl, passive pump: sponge, distance Inj-WE: 30 mm, Configuration D, DSA468.

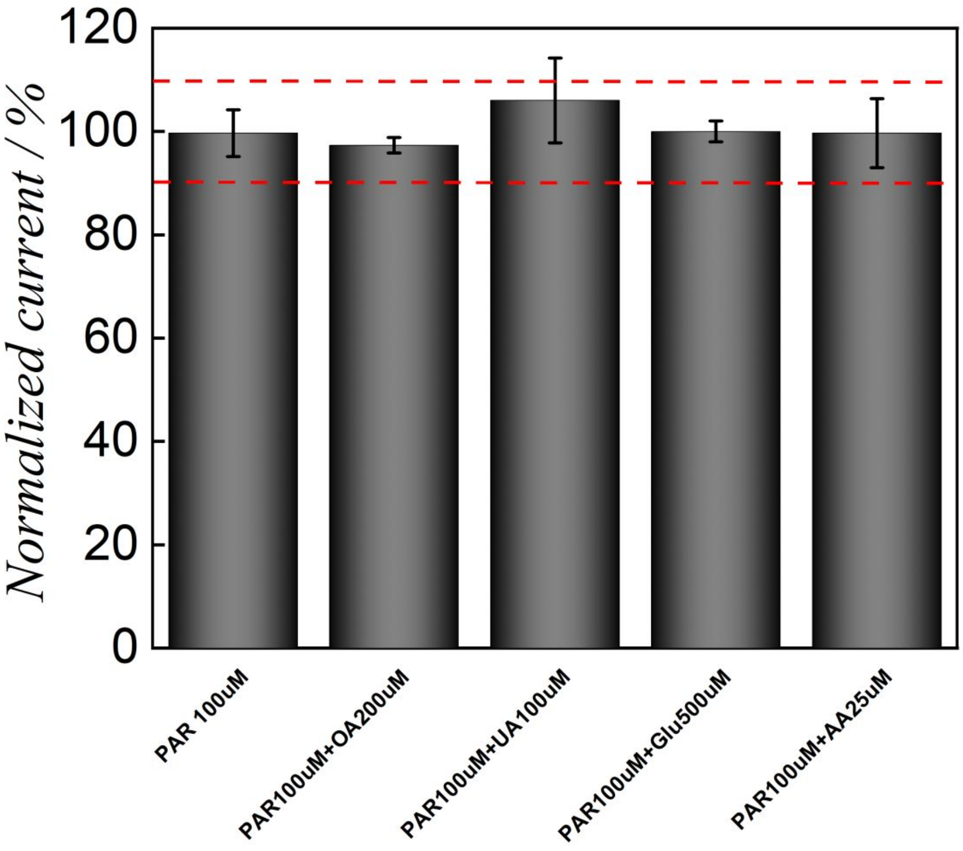

Figure S17. Effect of possible interfering species on the voltametric signal of BPA, presence of 100 µmol L−1 PAR: oxalic acid 200 µmol L−1, uric acid 100 µmol L−1, glucose 500 µmol L−1 and ascorbic acid 200 µmol L−1. Conditions: Egenerator = +0.90 V and Ecollector = -0.05 vs. Ag/AgCl, passive pump: sponge, distance Inj-WE: 30 mm, Configuration D, DSA468.

REFERENCES

(1) Jang, I.; Carrao, D. B.; Menger, R. F.; Moraes de Oliveira, A. R.; Henry, C. S. Pump-Free Microfluidic Rapid Mixer Combined with a Paper-Based Channel. *ACS Sens* **2020**, *5* (7), 2230–2238. DOI: 10.1021/acssensors.0c00937.
(2) Pradela-Filho, L. A.; Noviana, E.; Araujo, D. A. G.; Takeuchi, R. M.; Santos, A. L.; Henry, C. S. Rapid Analysis in Continuous-Flow Electrochemical Paper-Based Analytical Devices. *ACS Sens* **2020**, *5* (1), 274–281. DOI: 10.1021/acssensors.9b02298.
(3) Noviana, E.; Klunder, K. J.; Channon, R. B.; Henry, C. S. Thermoplastic Electrode Arrays in Electrochemical Paper-Based Analytical Devices. *Anal Chem* **2019**, *91* (3), 2431–2438. DOI: 10.1021/acs.analchem.8b05218.
(4) Kestin, J.; Khalifa, H. E.; Correia, R. J. Tables of the dynamic and kinematic viscosity of aqueous KCl solutions in the temperature range 25–150 °C and the pressure range 0.1–

35 MPa. *Journal of Physical and Chemical Reference Data* **1981**, *10* (1), 57–70. DOI: 10.1063/1.555640 (accessed 2/9/2026).
(5) Jang, I.; Kang, H.; Song, S.; Dandy, D. S.; Geiss, B. J.; Henry, C. S. Flow control in a laminate capillary-driven microfluidic device. *Analyst* **2021**, *146* (6), 1932–1939. DOI: 10.1039/d0an02279a.
